# Optimized scheduling of integrated energy systems considering waste-to-power plants and advanced adiabatic air compression energy storage machines

**DOI:** 10.1038/s41598-026-37485-z

**Published:** 2026-02-10

**Authors:** Weijian Wang, Min Liu, Haiqiang Zhao, Yuanda Wu, Yongyuan Tian

**Affiliations:** 1 College of Electrical Engineering, Guizhou University, Guiyang, Guizhou China; 2 Guizhou Provincial Key Laboratory of Power System Intelligent Technologies, Guiyang, China

**Keywords:** Waste incineration power plant, Ammonia-coal co-firing, Advanced adiabatic compressed air energy storage, Integrated energy system, Improved particle swarm algorithm, Energy science and technology, Engineering

## Abstract

To achieve carbon peaking and carbon neutrality goals, improve energy utilization efficiency, and accelerate the decarbonization of energy structure, this paper proposes a model that integrates Waste Incineration Power Plant (WIP) and Advanced Adiabatic Compressed Air Energy Storage (AA-CAES) to reduce carbon emissions and enhance system economics. First, based on the coupled WIP and Power-to-Gas (P2G) model, a waste heat recovery unit is introduced to recover exhaust heat and reduce purchase heat cost. Second, Power-to-Ammonia (P2A) technology is integrated with coal-fired generating units to enable dynamic ammonia-coal co-firing, further reducing carbon emissions and enhancing renewable energy utilization. Third, AA-CAES is incorporated to expand heat supply channels through compression heat storage and release, while absorbing heat during expansion power generation, thus achieving cross-temporal heat utilization and establishing a coordinated power and heat supply model between energy storage equipment and WIP. Finally, an improved Particle Swarm Optimization algorithm with dynamically adjusted inertia weights and learning factors, combined with a local exchange strategy, is employed for optimization. Case study results demonstrate that the proposed improved algorithm achieves lower total cost, and the coordinated operation of AA-CAES with WIP reduces the total system cost by 20.03%.

**Supplementary Information:**

The online version contains supplementary material available at 10.1038/s41598-026-37485-z.

## Introduction

In the context of the global energy crisis and climate change, energy structure transformation has become imperative^1^. To achieve carbon neutrality and sustainable development, countries worldwide are actively promoting emission reduction technologies and renewable energy development^2^. Integrated Energy Systems (IES), as a multi-energy complementary, multi-source synergistic energy utilization mode^3^, has gradually become an important way to solve energy and environmental problems^4^.

Waste-to-energy incineration technology not only addresses municipal waste issues effectively, but also enhances the feasibility of energy sustainability^5^. Literature^6^constructed an energy system containing a WIP and a coal-fired generator to improve the energy recovery rate of waste and reduce the investment cost of the WIP. P2G technology converts electrical energy into methane to supply steam-fired units, realizing energy conversion and consumption. Coupling P2G with incineration power plants captures CO_2_ in the flue gas and provides CO_2_ feedstock for the methanation reaction unit in the P2G equipment, reducing carbon emissions. Literature^7^ constructs a model of WIP that includes a flue gas cleaning system, a waste anaerobic fermentation facility for biogas purification, a carbon capture technology, a hydrogen diversification technology, and an electric-to-gas facility, which facilitates energy coupling and reduces carbon emissions and operating cost. Literature^8^ proposes a new virtual power plant architecture coupled with carbon capture for gas-fired power generation, electricity-to-gas conversion, and waste-to-energy incineration to facilitate a clean and low-carbon transition of the energy system.

Combining waste-to-energy incineration with waste temperature utilization technology not only improves the energy efficiency of the unit but is also important for achieving the dual-carbon goal^9^. Literature^10^ reused waste energy contained in the flue gas at the chimney outlet of a WIP to improve the district heating conversion efficiency. Literature^11^ applied absorption heat pumps to incineration power plants to realize waste heat recovery and improve the conversion efficiency of power generation. Ammonia produced by P2A technology, as a potential zero-carbon fuel, can effectively reduce carbon emissions from thermal power units by mixing with coal^12^. Literature^13^synthesized green hydrogen generated from wind and solar energy to supply green ammonia to thermal units for blending to reduce the cost of thermal power generation and carbon emissions. Most of the above studies have confirmed that P2G, waste temperature utilization and P2A technologies can reduce IES system cost and carbon emissions when combined with WIP, respectively, but fewer studies have examined the roles and mechanisms of the synergies between these three technologies and WIP on the operation of IES in a low-carbon economy.

In recent years, the emerging AA-CAES has attracted much attention due to its advantages, such as long lifetime, zero carbon emission, and low cost, and its natural cogeneration capability^14^. Literature^15^designed a new type of combined system of IES, AA-CAES, and organic Rankine cycle to realize the flexibility and low-carbon nature of the system’s energy supply, and the results of the study can provide a reference for the operation optimization of complex integrated energy systems. Literature^16^ establishes an optimal scheduling model of IES with AA-CAES as the energy hub, and introduces a stepwise carbon trading mechanism by combining its multi-energy characteristics, which verifies the effectiveness of the coupling of the two on the development of IES low-carbon economy. The above studies verified that AA-CAES has a low-carbon effect on the integrated energy system, but less research has been done on the effects of changes in the parameters of AA-CAES itself on the performance of AA-CAES and IES.

Particle swarm optimization (PSO), a widely used intelligent optimization algorithm, has been extensively applied to various engineering optimization problems due to its fast convergence and simple implementation^17^. For the non-convex economic dispatch problem involving valve point effects and multi-fuel selection, literature^18^ uses the improved integrated learning PSO based on forgetting speed to achieve an effective solution; literature^19^ proposes the improved competitive swarm optimization algorithm for solving the multi-area economic dispatch. The PSO algorithm is improved in the above literature and successfully applied in the field of power scheduling. Aiming to address the particle swarm algorithm’s shortcomings, such as its tendency to fall into local optima and the difficulty in adjusting inertia weights and learning factors^20^, this paper proposes improvements to enhance the algorithm’s performance.

Table 1 presents a literature review summary of the relevant field, which systematically sorts out the technical contributions of existing research in the directions of waste incineration power generation, integrated energy system coupling, and low-carbon dispatch optimization, and clearly points out the limitations of each study in terms of model development, technical mechanism interpretation, and algorithm adaptability.

**Table 1:** Literature review summary.

Ref	Research content	Advantages	Limitations
^6^	Constructed an energy system integrating waste incineration power generation and coal-fired generation	Improved the energy recovery rate of generating units	Insufficient model development; optimization operation of waste incineration power plant integrated energy systems has not been adequately studied
^7^	Developed a waste incineration power plant model incorporating flue gas purification system, anaerobic digestion facility for biogas purification, carbon capture technology, hydrogen diversification technology, and power-to-gas facilities	Achieved energy coupling, reduced carbon emissions and operating cost	
^10,11^	Integrated waste incineration power generation with waste heat recovery technology	Enhanced energy utilization efficiency	
^13^	Comprehensively utilized green hydrogen produced from wind and solar energy to provide green ammonia for co-firing in thermal power units to reduce fossil fuel power generation cost and carbon emissions	Improved economic benefits and achieved full utilization of system products	Insufficient discussion on the role and mechanism of power-to-gas and power-to-ammonia technologies in the coordinated operation of integrated energy systems
^15^	Combined advanced adiabatic compressed air energy storage (AA-CAES) with organic Rankine cycle	Achieved system energy supply flexibility and low-carbon characteristics, providing reference for operation optimization of complex integrated energy systems	Did not explore the impact of AA-CAES parameter variations on its performance and the integrated energy system
^16^	Established an optimal scheduling model for an integrated energy system with AA-CAES as the energy hub, and introduced a tiered carbon trading mechanism based on its multi-energy characteristics	Validated the effectiveness of their coupling for low-carbon economic development of integrated energy systems	
^18^	Improved ensemble learning particle swarm optimization algorithm based on forgetting rate to solve economic dispatch problems in power systems	Achieved effective solution and demonstrated the feasibility of PSO for solving power dispatch problems	The model in this paper involves multiple energy conversion devices participating in gas collection and conversion with more complex modeling, requiring further improvement of the PSO algorithm
^19^	Proposed an improved competitive swarm optimization algorithm for solving multi-area economic dispatch problems		

To address the aforementioned challenges, this paper proposes a low-carbon economic dispatch model for an integrated energy system (IES) based on waste incineration power plant (WIP) coupled with power-to-gas (P2G) technology. The model incorporates waste heat recovery and power-to-ammonia (P2A) for synergistic carbon reduction, as well as advanced adiabatic compressed air energy storage (AA-CAES) for coordinated power and heat supply with WIP. The low-carbon and economic performance of the proposed model is validated through numerical simulations. The main contributions are as follows:

1. WIP coupled with P2G integrated with waste heat recovery and P2A for synergistic carbon reduction: CO₂ captured by the carbon capture device is converted to methane through P2G technology, while N₂ separated by pressure swing adsorption (PSA) is utilized for ammonia synthesis via P2A. Meanwhile, waste heat is recovered by the heat recovery system, thereby achieving low-carbon energy supply for the integrated system.
2. Joint model of AA-CAES and WIP: The former stores electricity and produces heat, and cooperates with WIP to supply heat, optimizing the energy structure to reduce carbon and increase efficiency, with large storage capacity and high conversion efficiency, which can reduce the renewable energy curtailment rate and enhance the benefits of IES.
3. Impact of compressor inlet temperature on the performance of AA-CAES and IES: Increasing the temperature can enhance its heating capacity, and heating the inlet temperature can improve the economy of IES.
4. PSO improvement: In view of the complex constraints of the model, the weights and learning factors are dynamically adjusted, and the local exchange strategy is adopted, which improves the quality of the solution and the stability of the algorithm.

## Model framework and operational process

**Fig. 1:**
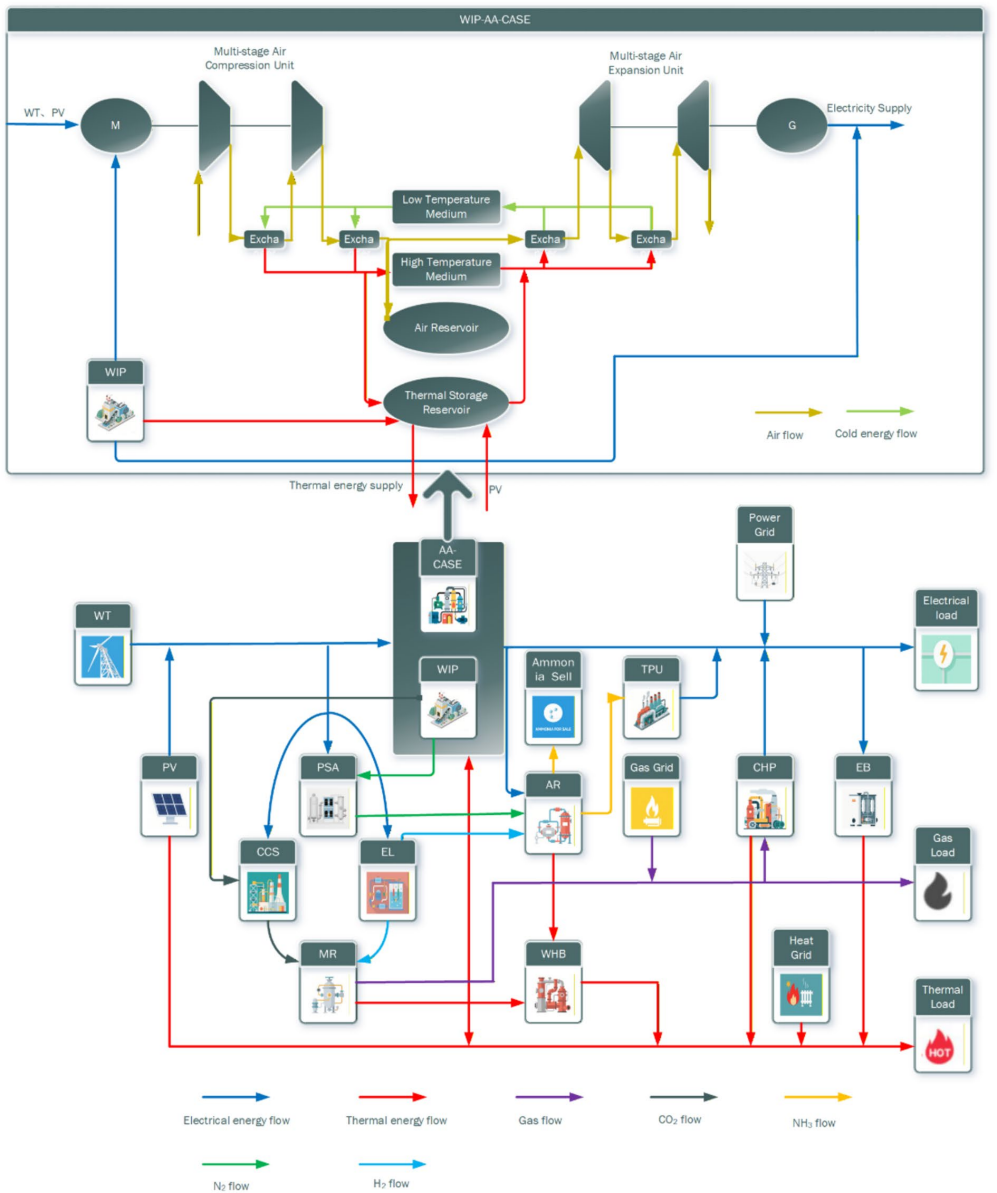
IES scheduling framework

The IES scheduling framework is illustrated in Fig. 1. The system serves electricity, heat, and gas loads. Electricity demand is met by waste incineration power plant (WIP), wind turbine (WT), photovoltaic (PV) system, combined heat and power (CHP) unit, thermal power unit (TPU), and the upper-level power grid. Heat demand is supplied by WIP, CHP units, waste heat boiler (WHB), electric boiler (EB), and the upper-level heat network. Energy conversion equipment includes ammonia reactor (AR), pressure swing adsorption (PSA) units, carbon capture and storage (CCS), electrolyzer (EL), and methanation reactor (MR). Energy storage is provided by advanced adiabatic compressed air energy storage (AA-CAES).

The operational process of the proposed model is as follows: The electrolyzer (EL) absorbs electrical energy to produce hydrogen (H₂). The gas generated from waste incineration power (WIP) undergoes treatment, after which carbon capture and storage (CCS) captures the CO₂. The captured CO₂ is then fed into the methanation reactor (MR) together with H₂ to produce methane (CH₄), which serves as fuel for the combined heat and power (CHP) unit. Simultaneously, pressure swing adsorption (PSA) adsorbs nitrogen (N₂) from the treated gas, and the N₂ is fed into the ammonia reactor (AR) along with H₂ to produce ammonia (NH₃). A portion of the produced NH₃ is used as fuel for the thermal power unit (TPU), while the remainder is sold commercially. The waste heat boiler (WHB) recovers thermal energy from WIP, MR, and AR, supplying it to thermal loads or feeding it into the advanced adiabatic compressed air energy storage (AA-CAES) system for expansion-based power generation. When renewable energy is abundant, the AA-CAES operates in compression mode, absorbing electrical energy while generating thermal energy for thermal loads. Conversely, when renewable energy is scarce, it operates in expansion mode, absorbing thermal energy to generate electrical energy for electrical loads.

Compared with traditional multi-energy complementary systems incorporating waste incineration power (WIP), this paper synthesizes ammonia from hydrogen for co-firing in thermal power units and commercial sale, thereby enhancing economic benefits and reducing carbon emissions. A waste heat boiler (WHB) is introduced to recover waste heat from WIP, P2G, and P2A units, optimizing energy utilization and conversion efficiency. To address the challenge that WIP’s electricity and heat supply cannot adequately meet power and heating demands, an advanced adiabatic compressed air energy storage (AA-CAES) system is integrated. The AA-CAES system enables flexible transitions between power supply and heat supply through compression for energy storage and expansion for power generation, better satisfying both electrical and thermal load requirements.

## Model composition

Restarting the unit requires additional cost and time^21^, and frequent startups and stops can cause irreversible mechanical damage to the unit, so startups and stops are not considered for the relevant generator and consumer units in this paper.

### Waste incineration power plant (WIP)model

#### WIP operational model

WIP can efficiently process large quantities of waste while reducing the consumption of non-renewable resources^22^.WIP has a constant daily volume of waste processed, and its associated modeling is as follows:

1 $$\left\{ \begin{gathered} M_{all}^{{{\mathrm{WIP}}}} = \sum\limits_{t = 1}^{24} {M_{t}^{{{\mathrm{WIP}}}} } ,P_{e,t}^{{{\mathrm{WIP}}}} = \eta_{e}^{{{\mathrm{WIP}}}} M_{t}^{{{\mathrm{WIP}}}} \hfill \\ P_{h,t}^{{{\mathrm{WIP}}}} = \eta_{h}^{{{\mathrm{WIP}}}} M_{t}^{{{\mathrm{WIP}}}} ,V_{t}^{{{\mathrm{WIP}}}} = \eta_{v}^{{{\mathrm{WIP}}}} M_{t}^{{{\mathrm{WIP}}}} \hfill \\ \end{gathered} \right.$$

where: $M_{all}^{{{\mathrm{WIP}}}}$ is the total amount of waste incinerated by the WIP per day, $M_{t}^{{{\mathrm{WIP}}}}$ is the amount of waste incinerated at time t, $P_{e,t}^{{{\mathrm{WIP}}}} ,P_{h,t}^{{{\mathrm{WIP}}}} ,V_{t}^{{{\mathrm{WIP}}}}$ are the electrical energy, heat energy and volume of exhaust gas produced by WIP at moment t, and $\eta_{e}^{{{\mathrm{WIP}}}} ,\eta_{h}^{{{\mathrm{WIP}}}} ,\eta_{v}^{{{\mathrm{WIP}}}}$ are the electrical energy, thermal energy, and exhaust gas generation conversion efficiency.

The WIP runs with the following constraints:

2 $$\left\{ \begin{gathered} P_{e,\min }^{{{\mathrm{WIP}}}} \le P_{e,t}^{{{\mathrm{WIP}}}} \le P_{e,\max }^{{{\mathrm{WIP}}}} \hfill \\ P_{h,\min }^{{{\mathrm{WIP}}}} \le P_{h,t}^{{{\mathrm{WIP}}}} \le P_{h,\max }^{{{\mathrm{WIP}}}} \hfill \\ \left| {P_{e,t}^{{{\mathrm{WIP}}}} - P_{e,t + 1}^{{{\mathrm{WIP}}}} } \right| \le \vartriangle P_{e}^{{{\mathrm{WIP}}}} \hfill \\ \end{gathered} \right.$$

where: $P_{e,\min }^{{{\mathrm{WIP}}}} ,P_{e,\max }^{{{\mathrm{WIP}}}}$ are the minimum and maximum WIP electrical energy output, $P_{h,\min }^{{{\mathrm{WIP}}}} ,P_{h,\max }^{{{\mathrm{WIP}}}}$ are the minimum and maximum WIP thermal energy output, and $\vartriangle P_{e}^{{{\mathrm{WIP}}}}$ is ramping power.

#### WIP flue gas treatment and waste heat recovery

Garbage in the incineration process discharges a large amount of flue gas containing sulfide, dioxin, particulate matter, and other harmful substances^23^, which needs to be sent to the flue gas purification device to be treated and qualified for discharge. Among them, the energy consumption of flue gas treatment accounts for about 1/4 of the total power generation^24^.

3 $$P_{e,t}^{{{\mathrm{FTA}}}} = \frac{{P_{e,t}^{{{\mathrm{WIP}}}} }}{4}$$

where $P_{e,t}^{{{\mathrm{FTA}}}}$ is the energy consumption of the WIP flue gas treatment unit.

After these treatments, the main remaining components in the flue gas are shown in Table 2^25^

**Table 2:** Flue gas composition.

Component	Value (%)
CO_2_	9.7 ± 3.1
N_2_	63.1 ± 6.6
H_2_O	17.1 ± 4.9
O_2_	10.1 ± 3.3

Based on the table, the CO_2_ and N_2_ emissions from the exhaust gases of the WIP can be calculated as

4 $$\left\{ \begin{gathered} M_{{{\mathrm{CO}}_{{2}} ,t}}^{{{\mathrm{WIP}}}} = 0.097V_{t}^{{{\mathrm{WIP}}}} \rho_{{{\mathrm{CO}}_{{2}} }} \hfill \\ M_{{{\mathrm{N}}_{{2}} ,t}}^{{{\mathrm{WIP}}}} = 0.631V_{t}^{{{\mathrm{WIP}}}} \rho_{{{\mathrm{N}}_{{2}} }} \hfill \\ \end{gathered} \right.$$

where $M_{{{\mathrm{CO}}_{{2}} ,t}}^{{{\mathrm{WIP}}}} ,M_{{{\mathrm{N}}_{{2}} ,t}}^{{{\mathrm{WIP}}}}$ are the mass of CO_2_ and N_2_ emitted by WIP at time t, and $\rho_{{{\mathrm{CO}}_{{2}} }} ,\rho_{{{\mathrm{N}}_{{2}} }}$ are the densities of CO_2_ and N_2_, respectively.

CO_2_ and N_2_ in the flue gas are separated using CCS and PSA equipment to deliver CO_2_ as feedstock to MR in P2G and N_2_ as feedstock to AR in P2A, respectively. The flue gas separation and waste heat recovery and utilization plant is modeled as follows:

5 $$\left\{ \begin{gathered} M_{{{\mathrm{CO}}_{{2}} {,}t}}^{{{\mathrm{CCS}}}} = \eta_{{{\mathrm{CO}}_{{2}} }} P_{t}^{{{\mathrm{CCS}}}} \hfill \\ M_{{{\mathrm{N}}_{{2}} ,t}}^{{{\mathrm{PSA}}}} = \eta_{{{\mathrm{N}}_{{2}} }} P_{t}^{{{\mathrm{PSA}}}} \hfill \\ P_{h,t}^{{{\mathrm{WHB}}}} = \eta_{h}^{{\mathrm{WHB,WIP}}} P_{h,t}^{{{\mathrm{WIP}}}} \hfill \\ \end{gathered} \right.$$

where $M_{{{\mathrm{CO}}_{{2}} ,t}}^{{{\mathrm{CCS}}}} ,M_{{{\mathrm{N}}_{{2}} ,t}}^{{{\mathrm{PSA}}}}$ are the mass of CO_2_ and N_2_ separated by the CCS and PSA equipment at time t, $P_{h,t}^{{{\mathrm{WHB}}}}$ is the heat recovered by the WHB at time t, $\eta_{{{\mathrm{CO}}_{{2}} }} ,\eta_{{{\mathrm{N}}_{{2}} }} ,\eta_{h}^{{\mathrm{WHB,WIP}}}$ are the CCS and PSA conversion efficiencies and heat recovery coefficients of the exhaust gas recovered by the WHB, and $P_{t}^{{{\mathrm{CCS}}}} ,P_{t}^{{{\mathrm{PSA}}}}$ are the power consumed by the CCS and PSA equipment at time t for electrical energy.

The constraints related to the CCS and PSA equipment are

6 $$\left\{ \begin{gathered} P_{\min }^{CCS} \le P_{t}^{CCS} \le P_{\max }^{CCS} ,P_{\min }^{PSA} \le P_{t}^{PSA} \le P_{\max }^{PSA} \hfill \\ \left| {P_{t}^{CCS} - P_{t + 1}^{CCS} } \right| \le \vartriangle P_{e}^{CCS} ,\left| {P_{t}^{PSA} - P_{t + 1}^{PSA} } \right| \le \vartriangle P_{e}^{PSA} \hfill \\ 0 \le M_{{CO_{2} ,t}}^{CCS} \le M_{{CO_{2} ,t}}^{WIP} ,0 \le M_{{N_{2} ,t}}^{PSA} \le M_{{N_{2} ,t}}^{WIP} \hfill \\ \end{gathered} \right.$$

where $P_{\min }^{{{\mathrm{CCS}}}} \left( {P_{\min }^{{{\mathrm{PSA}}}} } \right),P_{\max }^{{{\mathrm{CCS}}}} \left( {P_{\max }^{{{\mathrm{PSA}}}} } \right)$ stand for the minimum and maximum power consumed by the CCS(PSA), $\vartriangle P_{e}^{{{\mathrm{CCS}}}}$ is ramping power of CCS equipment, and $\vartriangle P_{e}^{{{\mathrm{PSA}}}}$ is ramping power of PSA equipment.

### Power-to-gas (P2G) model

P2G consists of two processes, electrolysis of water and hydrogen to methane, which can enhance the electric-gas coupling characteristics of the system^26^. In a methane reactor, hydrogen reacts with CO_2_ to produce methane and water through catalysis^27^.

(1) EL model

7 $$\left\{ \begin{gathered} P_{{{\mathrm{H}}_{{2}} ,t}}^{{{\mathrm{EL}}}} = \eta^{{{\mathrm{EL}}}} P_{e,t}^{{{\mathrm{EL}}}} \hfill \\ P_{\min }^{{{\mathrm{EL}}}} \le P_{e,t}^{{{\mathrm{EL}}}} \le P_{\max }^{{{\mathrm{EL}}}} \hfill \\ \end{gathered} \right.$$

where, $P_{{{\mathrm{H}}_{{2}} ,t}}^{{{\mathrm{EL}}}}$ is the H_2_ output of the EL at time t, $P_{e,t}^{{{\mathrm{EL}}}}$ is the power consumed by the EL at time t, $\eta_{{}}^{{{\mathrm{EL}}}}$ is the conversion efficiency of the EL device, and $P_{\min }^{{{\mathrm{EL}}}} ,P_{\max }^{{{\mathrm{EL}}}}$ stand for the minimum and maximum power consumed by the EL.

(2) MR model

8 $$\left\{ \begin{gathered} P_{{{\mathrm{CH}}_{{4}} ,t}}^{{{\mathrm{MR}}}} = \eta^{{{\mathrm{MR}}}} P_{{{\mathrm{H}}_{{2}} ,t}}^{{{\mathrm{MR}}}} \hfill \\ P_{{{\mathrm{H}}_{{2}} ,\min }}^{{{\mathrm{MR}}}} \le P_{{{\mathrm{H}}_{{2}} ,t}}^{{{\mathrm{MR}}}} \le P_{{{\mathrm{H}}_{{2}} ,\max }}^{{{\mathrm{MR}}}} \hfill \\ \left| {P_{{{\mathrm{H}}_{{2}} ,,t}}^{{{\mathrm{MR}}}} - P_{{{\mathrm{H}}_{{2}} ,,t + 1}}^{{{\mathrm{MR}}}} } \right| \le \vartriangle P_{{{\mathrm{H}}_{{2}} }}^{{{\mathrm{MR}}}} \hfill \\ \end{gathered} \right.$$

where, $P_{{{\mathrm{CH}}_{{4}} ,t}}^{{{\mathrm{MR}}}}$ is the CH_4_ output of the MR at time t, $P_{{{\mathrm{H}}_{{2}} ,t}}^{{{\mathrm{MR}}}}$ is the H_2_ consumed by the MR at time t, $\eta_{{}}^{{{\mathrm{MR}}}}$ is the conversion efficiency of the MR device, $P_{{{\mathrm{H}}_{{2}} ,\min }}^{{{\mathrm{MR}}}} ,P_{{{\mathrm{H}}_{{2}} ,\max }}^{{{\mathrm{MR}}}}$ stand for minimum and maximum power of H_2_ consumption by the MR, and $\vartriangle P_{{{\mathrm{H}}_{{2}} }}^{{{\mathrm{MR}}}}$ is ramping power of the MR device.

(3) WHB Recovery P2G Waste Heat Modeling

9 $$P_{{{\mathrm{CH}}_{{4}} ,h,t}}^{{{\mathrm{WHB}}}} = \eta_{h}^{{\mathrm{WHB,MR}}} \alpha_{{{\mathrm{CH}}_{{4}} }} P_{{{\mathrm{CH}}_{4} ,t}}^{{{\mathrm{MR}}}}$$

where $P_{{{\mathrm{CH}}_{{4}} ,h,t}}^{{{\mathrm{WHB}}}}$ is the thermal energy recovered by the WHB to produce CH_4_ at moment t, $\alpha_{{{\mathrm{CH}}_{4} }}$ is the thermal power released to produce a unit of CH_4_, and $\eta_{h}^{{\mathrm{WHB,MR}}}$ is the heat recovery coefficient of the WHB to recover P2G.

### Coal-ammonia co-firing model

#### Power-to-ammonia (P2A) model

Ammonia is a new energy fuel with great potential, and green ammonia consumes only 7.4 kWh of electricity to produce 1 kg of NH_3_^28^. The synthesis of ammonia is usually too slow, so catalysts must be used to increase the synthesis rate^29^. During ammonia synthesis, the heat generated by the reaction is typically dissipated directly into the atmosphere, causing a waste of resources. In order to minimize heat waste, WHB is used to recover and used to supply the thermal load.

(1) EL model.

See Eq. (7).

(2) AR model

10 $$\left\{ \begin{gathered} M_{{NH_{3} ,t}}^{AR} = \eta^{AT} P_{e,t}^{AR} ,M_{{N_{2} ,t}}^{AR} = M_{{N_{2} ,t}}^{PSA} \hfill \\ P_{e,\min }^{AR} \le P_{e,t}^{AR} \le P_{e,\max }^{AR} ,\left| {P_{e,t}^{AR} - P_{e,t + 1}^{AR} } \right| \le \vartriangle P_{e}^{AR} \hfill \\ \end{gathered} \right.$$

where $M_{{{\mathrm{NH}}_{{3}} ,t}}^{{{\mathrm{AR}}}}$ is the mass of NH_3_ generated by the AR at time t, $M_{{{\mathrm{N}}_{{2}} ,t}}^{{{\mathrm{AR}}}}$ is the mass of N_2_ consumed by the AR at time t, $P_{e,t}^{{{\mathrm{AR}}}}$ is the power consumed by the AR at time t, $\eta_{{}}^{{{\mathrm{AR}}}}$ is the conversion efficiency of the AR device, $P_{e,\min }^{{{\mathrm{AR}}}} ,P_{e,\max }^{{{\mathrm{AR}}}}$ are the minimum and maximum power of power consumed by the AR, and $\vartriangle P_{e}^{{{\mathrm{AR}}}}$ is ramping power of the AR device.

(3) WHB Recovery of P2A Waste Heat Modeling

11 $$P_{{{\mathrm{NH}}_{{3}} ,h,t}}^{{{\mathrm{WHB}}}} = \eta_{h}^{{\mathrm{WHB,AR}}} \alpha_{{{\mathrm{NH}}_{{3}} }} M_{{{\mathrm{NH}}_{{3}} ,t}}^{{{\mathrm{AR}}}}$$

where $P_{{{\mathrm{NH}}_{{3}} ,h,t}}^{{{\mathrm{WHB}}}}$ is the thermal energy recovered by the WHB to produce NH_3_ at moment t, $\alpha_{{{\mathrm{NH}}_{{3}} }}$ is the thermal power released to produce a unit of NH_3_, and $\eta_{h}^{{\mathrm{WHB,AR}}}$ is the heat recovery coefficient of the WHB to recover P2A.

#### Coal-ammonia Co-firing thermal power unit modeling

Ammonia-coal blending effectively lowers NOx emissions, thus serving as a suitable low-carbon alternative fuel for coal-fired units^30^.The upper limit of the fuel blending ratio of ammonia for TPU is taken as 20%^13^.

12 $$\left\{ \begin{gathered} M_{t}^{{\mathrm{TPU,coal}}} = c + bP_{t}^{{{\mathrm{TPU}}}} + a\left( {P_{t}^{{{\mathrm{TPU}}}} } \right)^{2} \hfill \\ P_{\min }^{{{\mathrm{TPU}}}} \le P_{t}^{{{\mathrm{TPU}}}} \le P_{\max }^{{{\mathrm{TPU}}}} ,\left| {P_{t}^{{{\mathrm{TPU}}}} - P_{t + 1}^{{{\mathrm{TPU}}}} } \right| \le \vartriangle P_{{}}^{{{\mathrm{TPU}}}} \hfill \\ M_{{{\mathrm{NH}}_{3} ,t}}^{{\mathrm{TPU,coal}}} = M_{t}^{{\mathrm{TPU,coal}}} - M_{{{\mathrm{NH}}_{{3}} ,t}} \frac{{L_{{{\mathrm{NH}}_{{3}} }} }}{{L_{{{\mathrm{coal}}}} }},k = \frac{{M_{{{\mathrm{NH}}_{{3}} ,t}} L_{{{\mathrm{NH}}_{{3}} }} }}{{M_{{{\mathrm{NH}}_{{3}} ,t}}^{{\mathrm{TPU,coal}}} L_{{{\mathrm{coal}}}} }} \hfill \\ M_{{{\mathrm{CO}}_{{2}} ,t}}^{{\mathrm{TPU,}}} = \eta_{{{\mathrm{CO}}_{{2}} }}^{{{\mathrm{TPU}}}} M_{{{\mathrm{NH}}_{3} ,t}}^{{\mathrm{TPU,coal}}} \hfill \\ \end{gathered} \right.$$

where $M_{t}^{{\mathrm{TPU,coal}}}$ is the coal consumption of TPU without ammonia at time t, $P_{t}^{{{\mathrm{TPU}}}}$ is the power output of TPU at time t, $P_{\min }^{{{\mathrm{TPU}}}} ,P_{{{\mathrm{max}}}}^{{{\mathrm{TPU}}}}$ are the minimum and maximum power output of TPU, $\vartriangle P_{{}}^{{{\mathrm{TPU}}}}$ is ramping power of TPU, $M_{{{\mathrm{NH}}_{{3}} ,t}}^{{\mathrm{TPU,coal}}}$ is the coal consumption of TPU with ammonia at time t, $M_{{{\mathrm{NH}}_{{3}} ,t}}^{{}}$ is the amount of ammonia doped by TPU at time t, $L_{{{\mathrm{NH}}_{{3}} }} ,L_{{{\mathrm{coal}}}}$ are the calorific value of ammonia and coal, $k$ is the ratio of ammonia doping, $M_{{{\mathrm{CO}}_{{2}} ,t}}^{{\mathrm{TPU,}}}$ is the carbon emission of TPU at time t, $\eta_{{{\mathrm{CO}}_{{2}} }}^{{{\mathrm{TPU}}}}$ is the carbon emission of each ton of coal. m is the carbon emission per ton of coal.

### Combined Heat and Power (CHP) Model

In this paper, the cogeneration unit is a back-pressure gas-fired unit, and the relationship equation between the power generation, heat generation and natural gas consumption of the gas-fired unit is as follows^31^

13 $$\left\{ \begin{gathered} P_{e,t}^{CHP} = \eta_{e}^{CHP} P_{{CH_{4} ,t}}^{CHP} ,P_{h,t}^{CHP} = \eta_{h}^{CHP} P_{E,t}^{CHP} \hfill \\ P_{e,\min }^{CHP} \le P_{e,t}^{CHP} \le P_{e,\max }^{CHP} ,\left| {P_{e,t}^{CHP} - P_{e,t + 1}^{CHP} } \right| \le \vartriangle P_{e}^{CHP} \hfill \\ \end{gathered} \right.$$

where $P_{e,t}^{{{\mathrm{CHP}}}} ,P_{{CH_{4} ,t}}^{{{\mathrm{CHP}}}}$ are the electrical energy output and CH_4_ power consumed by the CHP at time t, $\eta_{e}^{{{\mathrm{CHP}}}} ,\eta_{h}^{{{\mathrm{CHP}}}}$ are the CH4-to-electrical energy efficiency and electrical energy-to-thermal energy efficiency of the CHP, $P_{e,\min }^{{{\mathrm{CHP}}}} ,P_{e,\max }^{{{\mathrm{CHP}}}}$ are the minimum and maximum output of electrical energy of the CHP, and $\vartriangle P_{e}^{{{\mathrm{CHP}}}}$ is ramping power of the CHP.

### Electric boiler (EB) model

14 $$\left\{ \begin{gathered} P_{h,t}^{{{\mathrm{EB}}}} = \eta^{{{\mathrm{EB}}}} P_{e,t}^{{{\mathrm{EB}}}} \hfill \\ P_{e,\min }^{{{\mathrm{EB}}}} \le P_{e,t}^{{{\mathrm{EB}}}} \le P_{e,\max }^{{{\mathrm{EB}}}} \hfill \\ \left| {P_{e,t}^{{{\mathrm{EB}}}} - P_{e,t + 1}^{{{\mathrm{EB}}}} } \right| \le \vartriangle P_{e}^{{{\mathrm{EB}}}} \hfill \\ \end{gathered} \right.$$

where, $P_{h,t}^{{{\mathrm{EB}}}} ,P_{e,t}^{{{\mathrm{EB}}}}$ are the thermal energy output and electrical energy consumed by the EB at time t, $\eta^{{{\mathrm{EB}}}}$ is the EB electrical energy to thermal energy efficiency, $P_{e,\min }^{{{\mathrm{EB}}}} ,P_{e,\max }^{{{\mathrm{EB}}}}$ are the minimum and maximum electrical energy consumed by the EB, and $\vartriangle P_{e}^{{{\mathrm{EB}}}}$ is the EB electrical ramping power.

### Photothermal conversion model

Photovoltaic cells can only convert a portion of the incident solar energy into electrical energy, while the remainder is converted into thermal energy. By recovering this thermal energy to supply heating loads, the overall energy utilization efficiency can be significantly improved^32^.

15 $$P_{h,t}^{{{\mathrm{PV}}}} = \alpha^{{{\mathrm{PV}}}} P_{e,t}^{{{\mathrm{PV}}}}$$

where $P_{h,t}^{{{\mathrm{PV}}}} ,P_{e,t}^{{{\mathrm{PV}}}}$ are the thermal energy the electrical energy generated by the PV at time t, and $\alpha^{{{\mathrm{PV}}}}$ is the conversion efficiency.

### Advanced adiabatic compressed air energy storage (AA-CAES) model

In this paper, we consider the operational constraints of all major components of the AA-CAES and ignore the dynamic constraints of the AA-CAES, such as the limitations on the rate of climb/descent, since CAES can usually be switched from the minimum power output to the maximum power output within 1 h^33^.

The modeling assumptions for the analysis are: i) Ideal air is used; ii) The heat and pressure losses from the devices are negligible; iii) The temperature of the storage tanks is constant; iv) The compressor and expander inlet temperatures are rated; v) The thermal storage tanks are set up to be adiabatic.

#### AA-case power storage and generation module

AA-CAES achieves electrical power charging and discharging by compressing and expanding air, and its related charging and discharging model^34^ is as follows:

16 $$\left\{ \begin{gathered} P_{ch\arg e,e,t}^{AA - CASE} = M_{ch\arg e,t} \frac{\gamma }{\gamma - 1}R_{g} \sum\limits_{i = 1}^{{n_{1} }} {\frac{{T_{ch\arg e,in,i}^{AA - CASE} }}{{\eta_{ch\arg e,i} }}\left( {\left( {\beta_{ch\arg e,i} } \right)^{{\frac{\gamma }{\gamma - 1}}} - 1} \right)} \hfill \\ P_{disch\arg e,e,t}^{AA - CASE} = M_{disch\arg e,t} \frac{\gamma }{\gamma - 1}R_{g} \sum\limits_{i = 1}^{{n_{2} }} {T_{disch\arg e,in,i}^{AA - CASE} \eta_{disch\arg e,i} \left( {1 - \left( {\beta_{disch\arg e,i} } \right)^{{\frac{1 - \gamma }{\gamma }}} } \right)} \hfill \\ P_{ch\arg e,e,\min }^{AA - CASE} \le P_{ch\arg e,e,t}^{AA - CASE} \le P_{ch\arg e,e,\max }^{AA - CASE} ,P_{disch\arg e,e,\min }^{AA - CASE} \le P_{disch\arg e,e,t}^{AA - CASE} \le P_{disch\arg e,e,\max }^{AA - CASE} \hfill \\ \mu_{ch\arg e,t}^{AA - CASE} + \mu_{disch\arg e,t}^{AA - CASE} \le 1 \hfill \\ \end{gathered} \right.$$

where $P_{{{\mathrm{charge}},e,t}}^{{\text{AA - CASE}}} ,P_{{{\mathrm{discharge}},e,t}}^{{\text{AA - CASE}}}$ for the moment t compressor electric power and the expansion of electric power, $M_{{{\mathrm{charge}},t}} ,M_{{{\mathrm{discharge}},t}}$ for the moment t into the compressor and the expansion of the air mass, $T_{{{\mathrm{charge}},in,i}}^{{\text{AA - CASE}}} ,T_{{{\mathrm{discharge}},in,i}}^{{\text{AA - CASE}}}$ for the i-th stage of the compressor and the expansion of the inlet air temperature, $\eta_{{{\mathrm{charge,}}i}} ,\eta_{{{\mathrm{discharge}},i}}$ for the i-th stage of the compressor and the expansion of the efficiency, $n_{1} ,n_{2}$ for the number of compressor and expansion of the number of stages, $\beta_{{{\mathrm{charge}},i}} ,\beta_{{{\mathrm{discharge}},i}}$ for the i-th stage of the compressor rated compression ratio and the expansion of the rated expansion ratio, $\gamma ,R_{g}$ are the specific heat capacity of air and ideal gas coefficient, $P_{{{\mathrm{charge}},e,\min }}^{{\text{AA - CASE}}} \left( {P_{{{\mathrm{discharge,}}e,\min }}^{{\text{AA - CASE}}} } \right),P_{{{\mathrm{charge}},e,\max }}^{{\text{AA - CASE}}} \left( {P_{{{\mathrm{discharge}},e,\max }}^{{\text{AA - CASE}}} } \right)$ are the minimum and maximum electric power of the compressor(expander).

#### AA-CAES Thermal Storage Module

When AA-CAES is charged and discharged, the air pressure in the gas chamber changes due to heat exchange with the outside world, and the relevant model is as follows^34^ :

17 $$\left\{ \begin{gathered} Q_{t} = \frac{{R_{g} \gamma T^{{{\mathrm{ST}}}} }}{{V^{{{\mathrm{ST}}}} }}\left( {M_{{{\mathrm{charge}},t}} - M_{{{\mathrm{discharge}},t}} } \right) - \left( {\nu + \kappa \left| {M_{{{\mathrm{charge}},t}} - M_{{{\mathrm{discharge}},t}} } \right|^{0.8} } \right)\left( {T^{{{\mathrm{ST}}}} - T_{wall}^{{{\mathrm{ST}}}} } \right) \hfill \\ Q_{t}^{{{\mathrm{ST}}}} = Q_{0}^{{{\mathrm{ST}}}} + \sum\limits_{i = 1}^{t} {Q_{t} } ,Q_{\min }^{{{\mathrm{ST}}}} \le Q_{t}^{{{\mathrm{ST}}}} \le Q_{\max }^{{{\mathrm{ST}}}} \hfill \\ \end{gathered} \right.$$

where $Q_{t}$ is the rate of change of air pressure in the gas storage chamber at time t, $V^{{{\mathrm{ST}}}}$ is the volume of the gas storage chamber, $T^{{{\mathrm{ST}}}} ,T_{wall}^{{{\mathrm{ST}}}}$ are the temperature inside the chamber and the temperature of the chamber wall, $\nu ,\kappa$ are the heat transfer coefficients due to natural convection and forced convection, $Q_{t}^{{{\mathrm{ST}}}}$ is the air pressure of the chamber at time t, and $Q_{\min }^{{{\mathrm{ST}}}} ,Q_{\max }^{{{\mathrm{ST}}}}$ are the allowable minimum and maximum atmospheric pressures of the gas storage chamber.

The thermal power storage, heating power supply and storage tank models^33^ for the AA-CAES system are as follows:

18 $$\left\{ \begin{gathered} T_{ch\arg e,out,i}^{AA - CASE} = T_{ch\arg e,in,i}^{AA - CASE} \left( {\frac{{\left( {\beta_{ch\arg e,i} } \right)^{{\frac{\gamma }{\gamma - 1}}} - 1}}{{\eta_{ch\arg e,i} }} + 1} \right),T_{disch\arg e,out,i}^{AA - CASE} = T_{disch\arg e,in,i}^{AA - CASE} \left( {\eta_{disch\arg e,i} \left( {\left( {\beta_{disch\arg e,i} } \right)^{{\frac{1 - \gamma }{\gamma }}} - 1} \right) + 1} \right) \hfill \\ P_{ch\arg e,h,t}^{AA - CASE} = M_{ch\arg e,t} \xi c_{air} \left( {\sum\limits_{i = 1}^{{n_{1} }} {T_{ch\arg e,out,i}^{AA - CASE} } - n_{1} T_{cold} } \right),P_{disch\arg e,h,t}^{AA - CASE} = M_{disch\arg e,t} \xi c_{air} \left( {n_{2} T_{hot} - T^{ST} - \sum\limits_{i = 1}^{{n_{2} - 1}} {T_{ch\arg e,out,i}^{AA - CASE} } } \right) \hfill \\ H_{t}^{ST} = H_{t - 1}^{ST} + P_{ch\arg e,h,t}^{AA - CASE} - P_{disch\arg e,h,t}^{AA - CASE} + H_{change,t}^{ST} \hfill \\ H_{\min }^{ST} \le H_{t}^{ST} \le H_{\max }^{ST} ,H_{change,\min }^{ST} \le H_{change,t}^{ST} \le H_{change,\max }^{ST} \hfill \\ \end{gathered} \right.$$

where $T_{{{\mathrm{charge,}}out,i}}^{{\text{AA - CASE}}} ,T_{{{\mathrm{discharge}},out,i}}^{{\text{AA - CASE}}}$ for the i-th stage compressor and expander outlet air temperature, $P_{{{\mathrm{charge}},h,t}}^{{\text{AA - CASE}}} ,P_{{{\mathrm{discharge}},h,t}}^{{\text{AA - CASE}}}$ for the moment t compression heat power release and expansion of absorbed heat power, $\xi ,c_{air}$ for the heat transfer coefficient and the isobaric heat capacity of the air, $T_{cold} ,T_{hot}$ for the low and high temperature heat transfer medium temperature, $H_{t}^{{{\mathrm{ST}}}} ,H_{{{\mathrm{change}},t}}^{{{\mathrm{ST}}}}$ for the heat storage tank in the moment of the t time of the heat storage and heat exchanged with the outside world.

## Constraints and objective function

### Power constraints

The inequality constraints in this paper include output and ramping constraints for each unit as specified in Eqs. 2 through 18. The electrical, thermal, natural gas, ammonia, carbon dioxide and hydrogen balances are constrained as follows:

19 $$\left\{ \begin{gathered} P_{together,e,t} = P_{disch\arg e,e,t}^{AA - CASE} + P_{e,t}^{WIP} \hfill \\ P_{together,e,t} + P_{t}^{TPU} + P_{e,t}^{CHP} + P_{e,t}^{BUY} + P_{t}^{WT} + P_{e,t}^{PV} \hfill \\ = P_{e,t}^{LOAD} + P_{t}^{CCS} + P_{t}^{PSA} + P_{t}^{EL} + P_{e,t}^{EB} + P_{e,t}^{AR} \hfill \\ P_{together,h,t} = P_{ch\arg e,h,t}^{AA - CASE} + P_{h,t}^{WIP} + H_{change,t}^{ST} \hfill \\ P_{together,h,t} + P_{h,t}^{CHP} + P_{h,t}^{BUY} + P_{{NH_{3} ,h,t}}^{WHB} + P_{{CH_{4} ,h,t}}^{WHB} + P_{h,t}^{PV} = P_{h,t}^{LOAD} \hfill \\ P_{{CH_{4} ,t}}^{MR} + P_{{CH_{4} ,t}}^{BUY} = P_{{CH_{4} ,t}}^{LOAD} + P_{{CH_{4} ,t}}^{CHP} ,P_{{H_{2} ,t}}^{EL} = P_{{H_{2} ,t}}^{MR} + P_{{H_{2} ,t}}^{AR} \hfill \\ M_{{NH_{3} ,t}}^{AR} = M_{{NH_{3} ,t}}^{TPU} + M_{t}^{Sell} ,M_{{CO_{2} ,t}}^{CCS} = M_{{CO_{2} ,t}}^{MR} + M_{{CO_{2} ,t}}^{K} \hfill \\ \end{gathered} \right.$$

where $P_{e,t}^{{{\mathrm{BUY}}}} ,P_{h,t}^{{{\mathrm{BUY}}}}$ are the purchased electric power and purchased thermal power at time t, $P_{t}^{{{\mathrm{WT}}}} ,P_{t}^{{{\mathrm{PV}}}}$ are the wind power and photovoltaic power at time t, $P_{e,t}^{{{\mathrm{LOAD}}}} ,P_{h,t}^{{{\mathrm{LOAD}}}} ,P_{{CH_{4} ,t}}^{{{\mathrm{LOAD}}}}$ are the electric, thermal, and gas loads at time t, $P_{{{\mathrm{CH}}_{{4}} ,t}}^{{{\mathrm{BUY}}}}$ is the purchased gas power at time t, and $M_{{{\mathrm{CO}}_{{2}} ,t}}^{{{\mathrm{MR}}}} ,M_{{{\mathrm{CO}}_{{2}} ,t}}^{{\mathrm{K}}}$ are the mass of CO_2_ consumed by the MR and the mass of CO_2_ sequestered at time t.

### Model variables

The optimization variables in this paper are the output of each unit for each time period, including: $$\begin{gathered} M_{t}^{WIP} ,P_{t}^{CCS} ,P_{t}^{PSA} ,P_{e,t}^{EL} ,P_{{H_{2} ,t}}^{MR} ,P_{e,t}^{AR} ,P_{t}^{TPU} ,P_{e,t}^{CHP} ,P_{e,t}^{EB} ,P_{e,t}^{PV} + P_{e,t}^{WT} ,P_{h,t}^{PV} , \hfill \\ H_{change,t}^{ST} ,P_{ch\arg e,e}^{AA - CASE} \left( {P_{disch\arg e,e}^{AA - CASE} } \right),P_{h,t}^{BUY} ,P_{e,t}^{BUY} \hfill \\ \end{gathered}$$, Total: 15 × 24 = 360.

### Total operation cost of IES

The optimization objective function of this paper is the total operation cost of IES, which includes the cost of energy purchase, carbon sequestration cost, equipment operation and maintenance cost, carbon trading cost, thermal power unit cost, and renewable energy cost.

20 $$\left\{ \begin{gathered} \min C = C^{{{\mathrm{BUY}}}} + C^{{\mathrm{K}}} + C^{{{\mathrm{IES}}}} + C_{{CO_{2} }}^{{{\mathrm{TRADE}}}} + C^{{{\mathrm{TPU}}}} + C^{{\mathrm{A}}} - C^{{{\mathrm{NH}}_{{3}} }} \hfill \\ C^{{{\mathrm{BUY}}}} = \sum\limits_{t = 1}^{24} {\left( {\alpha_{e}^{{{\mathrm{BUY}}}} P_{e,t}^{{{\mathrm{BUY}}}} + \alpha_{h}^{{{\mathrm{BUY}}}} P_{h,t}^{{{\mathrm{BUY}}}} + \alpha_{{{\mathrm{CH}}_{{4}} }}^{{{\mathrm{BUY}}}} P_{{{\mathrm{CH}}_{{4}} ,t}}^{{{\mathrm{BUY}}}} } \right)\vartriangle t} \hfill \\ C^{{\mathrm{K}}} = \sum\limits_{t = 1}^{24} {\left( {\alpha_{{{\mathrm{CO}}_{{2}} }}^{{\mathrm{K}}} M_{{{\mathrm{CO}}_{{2}} {,}t}}^{{\mathrm{K}}} } \right)\vartriangle t,C^{{{\mathrm{TPU}}}} = \sum\limits_{t = 1}^{24} {\left( {\alpha_{{}}^{{{\mathrm{coal}}}} M_{{{\mathrm{NH}}_{3} ,t}}^{{\mathrm{TPU,coal}}} + \alpha_{{}}^{{{\mathrm{EN}}}} M_{{{\mathrm{NH}}_{{3}} ,t}}^{{\mathrm{TPU,coal}}} } \right)\vartriangle t} } \hfill \\ C^{{\mathrm{A}}} = \sum\limits_{t = 1}^{24} {\alpha_{{}}^{{\mathrm{A}}} \left( {P_{\max ,t}^{{{\mathrm{WT}}}} + P_{\max ,t}^{{{\mathrm{PV}}}} - P_{t}^{{{\mathrm{WT}}}} - P_{t}^{{{\mathrm{PV}}}} } \right)\vartriangle t} \hfill \\ \end{gathered} \right.$$

where $C,C^{{{\mathrm{BUY}}}} ,C^{{\mathrm{K}}} ,C^{{{\mathrm{IES}}}} ,C_{{{\mathrm{CO}}_{{2}} }}^{{{\mathrm{TRADE}}}} ,C^{{{\mathrm{TPU}}}} ,C^{{\mathrm{A}}} ,C^{{{\mathrm{NH}}_{{3}} }}$ are the total cost, purchased energy cost, carbon sequestration cost, equipment cost, carbon trading cost, thermal power unit cost, renewable energy cost and profit from selling ammonia, $\alpha_{e}^{{{\mathrm{BUY}}}} ,\alpha_{h}^{{{\mathrm{BUY}}}} ,\alpha_{{{\mathrm{CH}}_{{4}} }}^{{{\mathrm{BUY}}}}$ are the price coefficients of purchased electricity, heat and gas, and $\alpha_{{{\mathrm{CO}}_{{2}} }}^{{\mathrm{K}}} ,\alpha_{{}}^{{{\mathrm{coal}}}} ,\alpha_{{}}^{{{\mathrm{EN}}}}$ are the coefficient of carbon sequestration cost, the price coefficient of coal combustion and the coefficient of environmental cost.

#### Equipment cost

21 $$\left\{ \begin{gathered} C^{{{\mathrm{IES}}}} = C^{{{\mathrm{Invest}}}} + C^{{{\mathrm{Run}}}} \hfill \\ C^{{{\mathrm{Invest}}}} = \frac{1}{365}\left( \begin{gathered} \alpha_{1}^{{\text{AA - CASE}}} \left( {P_{ch\arg e,e,\max }^{{\text{AA - CASE}}} + P_{disch\arg e,e,\max }^{{\text{AA - CASE}}} } \right) + \alpha_{2}^{{\text{AA - CASE}}} V^{{{\mathrm{ST}}}} + \hfill \\ \alpha_{3}^{{\text{AA - CASE}}} H_{\max }^{{{\mathrm{ST}}}} + \alpha_{{}}^{{{\mathrm{EL}}}} P_{e,\max }^{{{\mathrm{EL}}}} + \alpha_{{}}^{{{\mathrm{PSA}}}} P_{\max }^{{{\mathrm{PSA}}}} + \alpha_{{}}^{{{\mathrm{MR}}}} P_{\max }^{{{\mathrm{MR}}}} \hfill \\ + \alpha_{{}}^{{{\mathrm{AR}}}} P_{e,\max }^{{{\mathrm{AR}}}} + \alpha_{{}}^{{{\mathrm{WHB}}}} P_{\max }^{{{\mathrm{WHB}}}} \hfill \\ \end{gathered} \right)\frac{{r\left( {1 + r} \right)^{40} }}{{\left( {1 + r} \right)^{40} - 1}} \hfill \\ C^{{{\mathrm{Run}}}} = \alpha^{{\mathrm{R}}} \left( {P_{{{\mathrm{charge}},e,\max }}^{{\text{AA - CASE}}} + P_{{{\mathrm{discharge}},e,\max }}^{{\text{AA - CASE}}} + P_{e,\max }^{{{\mathrm{EL}}}} + P_{\max }^{{{\mathrm{PSA}}}} + P_{\max }^{{{\mathrm{MR}}}} + P_{e,\max }^{{{\mathrm{AR}}}} + P_{\max }^{{{\mathrm{WHB}}}} } \right) \hfill \\ \end{gathered} \right.$$

where: $C^{Invest} ,C^{Run}$ are the average daily construction cost and equipment maintenance cost, $r$ is the discount rate, $\alpha_{1}^{{\text{AA - CASE}}} ,\alpha_{2}^{{\text{AA - CASE}}} ,\alpha_{3}^{{\text{AA - CASE}}}$ are the construction cost per unit of compressor (expansion) power, construction cost per unit of storage room volume and construction cost per unit of storage tank capacity, $\alpha_{{}}^{{{\mathrm{EL}}}} ,\alpha_{{}}^{{{\mathrm{PSA}}}} ,\alpha_{{}}^{{{\mathrm{MR}}}} ,\alpha_{{}}^{{{\mathrm{AR}}}} ,\alpha_{{}}^{{{\mathrm{WHB}}}} ,\alpha_{{}}^{{\mathrm{R}}}$ are the construction cost per unit of EL(PSA,MR,AR,WHB) power, and equipment unit power maintenance cost factor.

##### Carbon trading cost

Carbon market trading mechanism, using the ladder carbon trading model^16^, the model is as follows:

22 $$\begin{gathered} E_{1} = \sum\limits_{i = 1}^{24} {\left( {M_{{{\mathrm{CO}}_{{2}} ,t}}^{{{\mathrm{TPU}}}} + M_{{{\mathrm{CO}}_{{2}} ,t}}^{{{\mathrm{WIP}}}} - M_{{{\mathrm{CO}}_{{2}} ,t}}^{{{\mathrm{CCS}}}} + \beta^{{{\mathrm{CHP}}}} P_{e,t}^{{{\mathrm{CHP}}}} + \beta_{e}^{{{\mathrm{BUY}}}} P_{e,t}^{{{\mathrm{BUY}}}} + \beta_{h}^{{{\mathrm{BUY}}}} P_{h,t}^{{{\mathrm{BUY}}}} } \right)\vartriangle t} \hfill \\ E_{2} = \sum\limits_{i = 1}^{24} {\left( {E^{{{\mathrm{TPU}}}} P_{t}^{{{\mathrm{TPU}}}} + E^{{{\mathrm{WTE}}}} P_{e,t}^{{{\mathrm{WTE}}}} + E^{{{\mathrm{CHP}}}} P_{e,t}^{{{\mathrm{CHP}}}} + E_{e}^{{{\mathrm{BUY}}}} P_{e,t}^{{{\mathrm{BUY}}}} + E_{h}^{{{\mathrm{BUY}}}} P_{h,t}^{{{\mathrm{BUY}}}} } \right)\vartriangle t} \hfill \\ E_{3} = E_{1} - E_{2} \hfill \\ C_{{{\mathrm{CO}}_{{2}} }}^{{{\mathrm{TRADE}}}} = \left\{ \begin{gathered} - \lambda \left( {2 + 3\delta } \right)l + \lambda \left( {1 + 3\delta } \right)\left( {E_{3} + 2l} \right),E_{3} \le - 2l \hfill \\ - \lambda \left( {1 + \delta } \right)l + \lambda \left( {1 + 2\delta } \right)\left( {E_{3} + l} \right), - 2l < E_{3} \le - l \hfill \\ \lambda \left( {1 + \delta } \right)E_{3} , - l < E_{3} \le 0 \hfill \\ \lambda E_{3} ,0 < E_{3} \le l \hfill \\ \lambda l + \lambda \left( {1 + \delta } \right)\left( {E_{3} - l} \right),l < E_{3} \le 2l \hfill \\ \lambda \left( {2 + \delta } \right)l + \lambda \left( {1 + 2\delta } \right)\left( {E_{3} - 2l} \right),2l < E_{3} \hfill \\ \end{gathered} \right. \hfill \\ \end{gathered}$$

where: $E_{1} ,E_{2} ,E_{3}$ are the actual carbon emissions, carbon emission allowances and the actual carbon trading volume participating in the carbon market, $\beta^{{{\mathrm{CHP}}}} ,\beta_{e}^{{{\mathrm{BUY}}}} ,\beta_{h}^{{{\mathrm{BUY}}}}$ are the CO_2_ emission factors of CHP units, purchased electricity and purchased heat, $E^{{{\mathrm{TPU}}}} ,E^{{{\mathrm{WTE}}}} ,E^{{{\mathrm{CHP}}}} ,E_{e}^{{{\mathrm{BUY}}}} ,E_{h}^{{{\mathrm{BUY}}}}$ are the carbon emission allowances of CHP, WIP, CHP, purchased electricity and purchased heat, $\lambda$ is the baseline price, $\delta$ is the growth coefficient, and $l$ is the step interval.

## Model solution

### Inertia weighting and learning factor improvement

To improve the search and learning ability of the particle swarm algorithm, the inertia weights and learning factors are improved using the dynamic adjustment of the literature^35^ with the following formulas:

23 $$\left\{ \begin{gathered} w\left( t \right) = w_{\max } - \sin \left[ {\frac{\pi }{2}\sqrt {\frac{t}{{t_{\max } }}} } \right]\left( {w_{\max } - w_{\min } } \right) \hfill \\ c_{1} \left( t \right) = \left( {c_{1f} - c_{1i} } \right)\frac{t}{{t_{\max } }} + c_{1i} ,c_{2} \left( t \right) = \left( {c_{2f} - c_{2i} } \right)\frac{t}{{t_{\max } }} + c_{2i} \hfill \\ \end{gathered} \right.$$

where: $w_{\max } ,w_{\min }$ are the maximum and minimum inertia weights, $t,t_{\max }$ are the current iteration and the maximum number of iterations, $c_{1i} ,c_{2i}$ are the initial learning factors, and $c_{1f} ,c_{2f}$ are the final learning factors.

### Local exchange

**Fig. 2:**
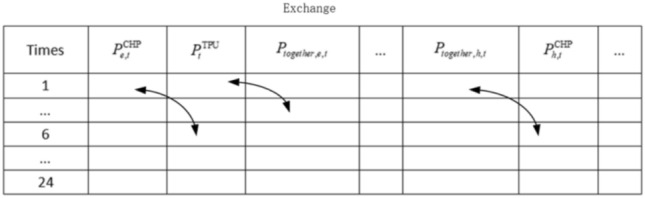
Schematic diagram of local exchange

In order to improve the efficiency of local search, the top 20% of the optimal values of the population are taken after global evolution and simulated annealing one by one (see Fig. 2 for the exchange schematic): the target individuals are selected and randomly exchanged for the allocation of the unit outputs in different time periods during the scheduling cycle, and the values of the decision variables are obtained by the constraints; the adaptive values of the two individuals are computed and compared, and the larger one is retained.

### Model framework and solution methodology

This chapter primarily focuses on the improvement of PSO, which features the following characteristics:

1. Dual Termination Mechanism: The algorithm incorporates two termination checks to ensure efficient convergence and timely termination when the maximum iteration limit is reached.
2. Constraint Handling Strategy: A normalization-based constraint handling approach is implemented to ensure all particles remain feasible throughout the optimization process. The iterative normalization loop guarantees compliance with Eqs. (1)-(19).
3. Local Exchange Operator: The integration of a local exchange operation enhances the algorithm’s exploitation capability by selecting and preserving superior individuals based on their fitness values.
4. Adaptive Parameter Adjustment: Dynamic parameter updating according to Eq. (23) enables the algorithm to adaptively balance exploration and exploitation during the search process.

**Fig. 3:**
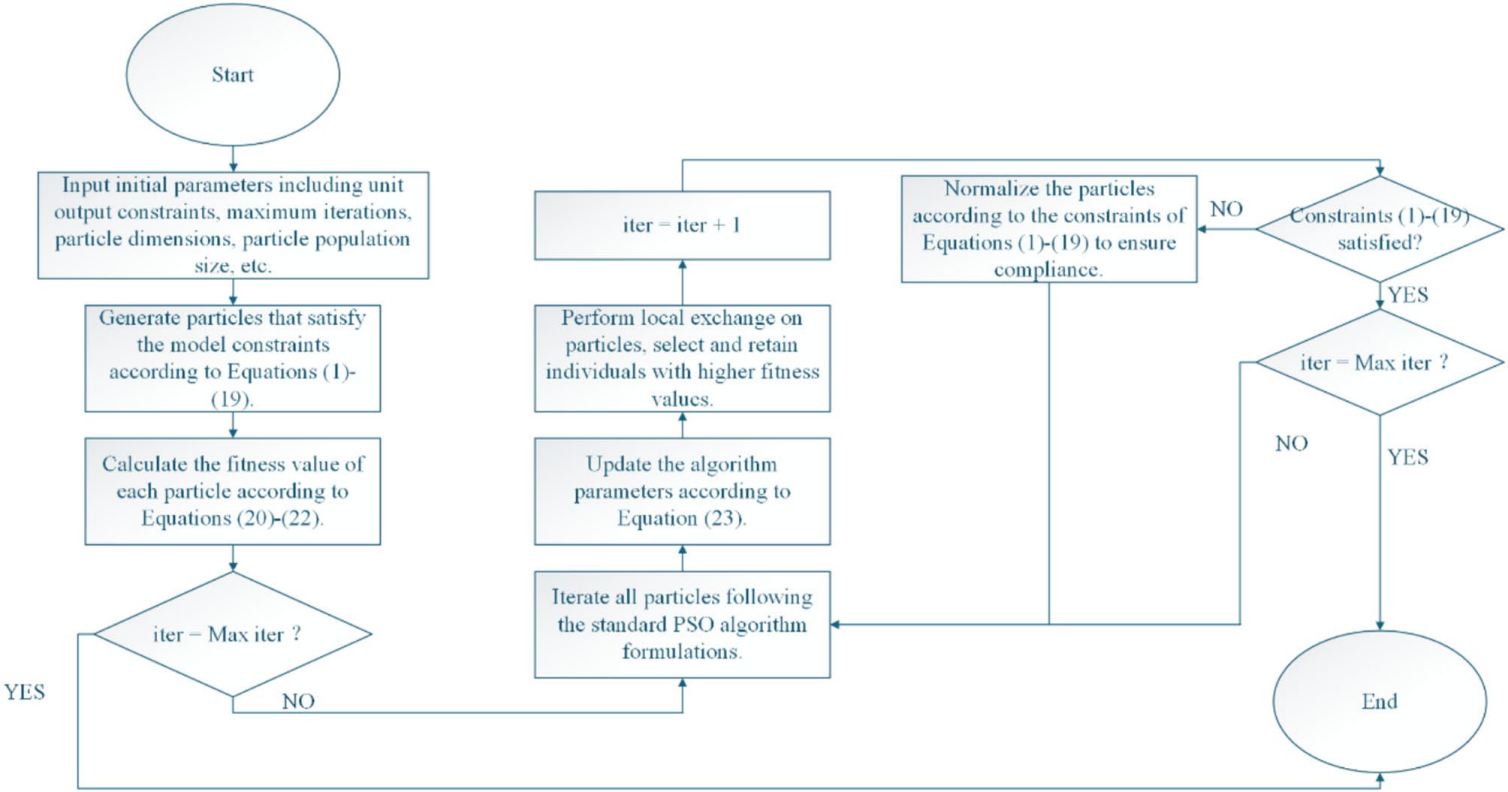
Flowchart

The main flowchart of the algorithm is shown in Fig. 3.

## Calculus analysis

**Fig. 4:**
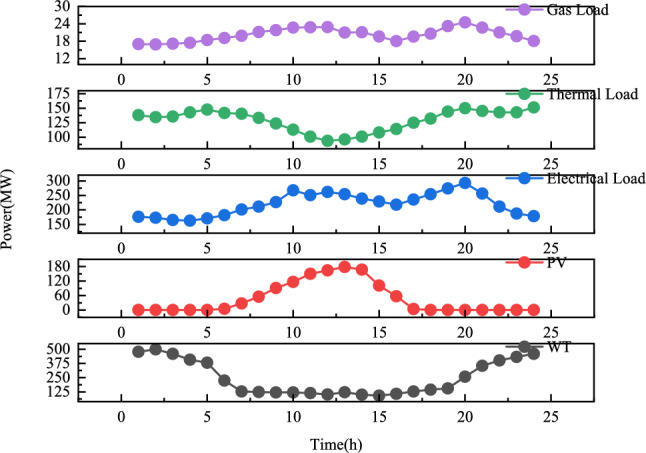
Forecast curves for gas load, thermal load, electricity load, light, and wind energy

The parameters of each unit are shown in Table A1, and the other parameters are shown in Table A2. Figure 4 shows the electric, thermal, and gas load data and the wind and solar output data.

### Scenario setting and analysis

To analyze the economy, low carbon and renewable energy consumption capacity of WHB, P2A, and AA-CASE for IES, four scenarios are set up, and Scenario 1 is the base scenario. Table 3 details the scenario settings and clearly presents the corresponding model configuration schemes for different scenarios.

**Table 3:** Scene settings.

	WIP-P2G	WHB	P2A	AA-CASE
Scenario 1	√			
Scenario 2	√	√		
Scenario 3	√	√	√	
Scenario 4	√	√	√	√

#### Comparison of different algorithms for solving

**Fig. 5:**
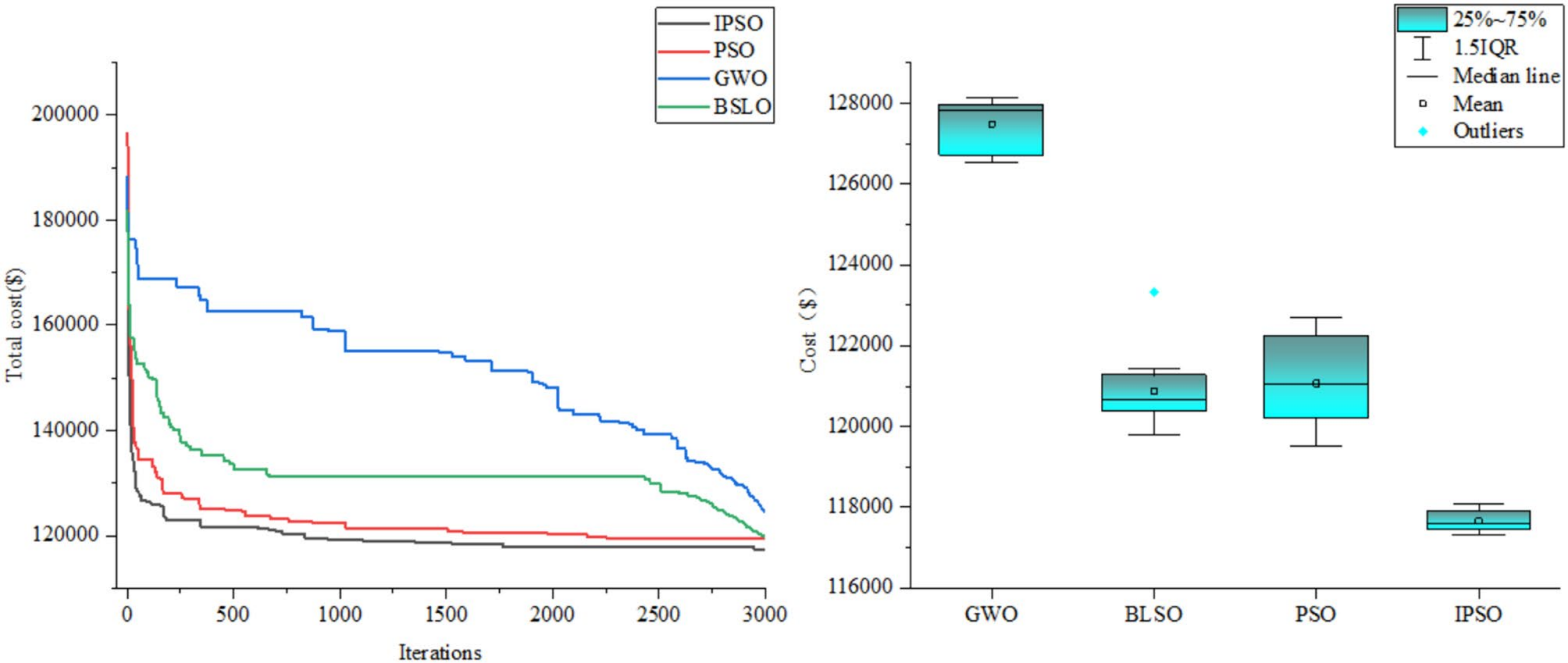
Comparison of algorithm solution results

Take scene 4 as an example, to verify the effectiveness of the improvement measures proposed in this paper, the improved particle swarm algorithm (IPSO), and PSO, the popular optimization algorithm Gray Wolf Algorithm^36^ (GWO) and the newest optimization algorithm Blood Sucking Leeches Optimization Algorithm^37^(BSLO) are compared, and the solution results are shown in Fig. 5.

As illustrated in Fig. 5, the proposed IPSO algorithm demonstrates superior accuracy and stability in solving the optimization model presented in this study. Compared with the conventional PSO algorithm, the IPSO exhibits faster convergence characteristics and achieves a lower final convergence value, thereby validating the rationality and superiority of the proposed improvements.

#### Scene scheduling results

**Fig. 6:**
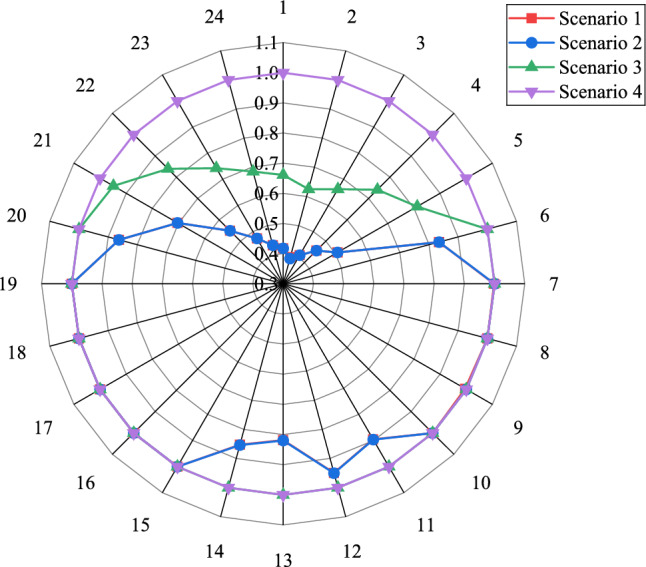
New energy utilization rate

The results of the algorithm solution are shown in Table 4, and Fig. 6 shows the comparison of 24 h scenery utilization for each scenario.

**Table 4:** Solution data for each scenario.

	Scenario 1	Scenario 2	Scenario 3	Scenario 4
Renewable energy cost ($)	22,476.0	22,476.0	10,198.54	0
Carbon trading cost($)	23,147.82	18,844.28	17,014.49	14,890.06
Purchased heat cost ($)	55,016.05	33,570.19	30,760.76	0.00
Purchased electricity cost ($)	105.57	109.62	1798.76	516.31
Purchase gas cost ($)	25,474.06	25,033.38	26,828.50	24,328.50
Carbon sequestration cost ($)	6445.63	6442.64	5157.76	695.20
Equipment cost ($)	6621.92	10,459.95	16,246.48	40,355.95
Thermal unit cost ($)	48,809.50	48,751.81	47,292.29	46,581.79
Profit from selling ammonia ($)	0.00	0.00	8588.34	10,052.45
Total cost ($)	188,096.52	165,687.95	146,709.27	117,315.38
Total thermal unit output (MWH)	880.48	877.63	1027.99	1011.79
Coal consumption(t)	595.24	594.53	576.84	568.02
Carbon sequestration (t)	214.85	214.75	171.92	23.17
Carbon emissions (t)	5711.90	5351.62	5337.79	4932.87
New energy utilization rate	68.02%	68.02%	85.49%	100.00%

This study comparatively analyzes the cost-effectiveness and environmental impacts across four operational scenarios, with the following key findings:

Economic Performance: In terms of total cost, Scenario 4 demonstrates optimal performance with a total cost of 117,315.38$.Carbon trading costs are highest in Scenario 1 and decrease to 14,890.06$ in Scenario 4, representing a 35.7% reduction. Purchased heat costs progressively decline from 55,016.05$ in Scenario 1, achieving zero external heat procurement in Scenario 4. Although equipment investment costs are highest in Scenario 4, they are partially offset by ammonia product sales revenue.

Environmental Performance: Scenario 4 exhibits superior environmental performance, with the lowest carbon emissions of only 4,932.87 tons, representing a 13.6% reduction compared to Scenario 1. The renewable energy utilization rate reaches 100%, significantly exceeding the 68.02% observed in Scenarios 1 and 2. Coal consumption is minimized at 568.02 tons, achieving a 4.6% reduction relative to Scenario 1.

Scenario 4 achieves a win–win outcome in both economic and environmental benefits through enhanced renewable energy utilization, optimized energy structure, and the integration of ammonia production. Despite higher initial capital investment in equipment, the scenario ultimately realizes optimal comprehensive benefits by reducing external energy procurement costs, minimizing carbon trading expenditures, and creating new revenue streams, thereby providing a viable technical pathway for low-carbon energy transition.

### Scheduling analysis under different scenarios

**Fig. 7:**
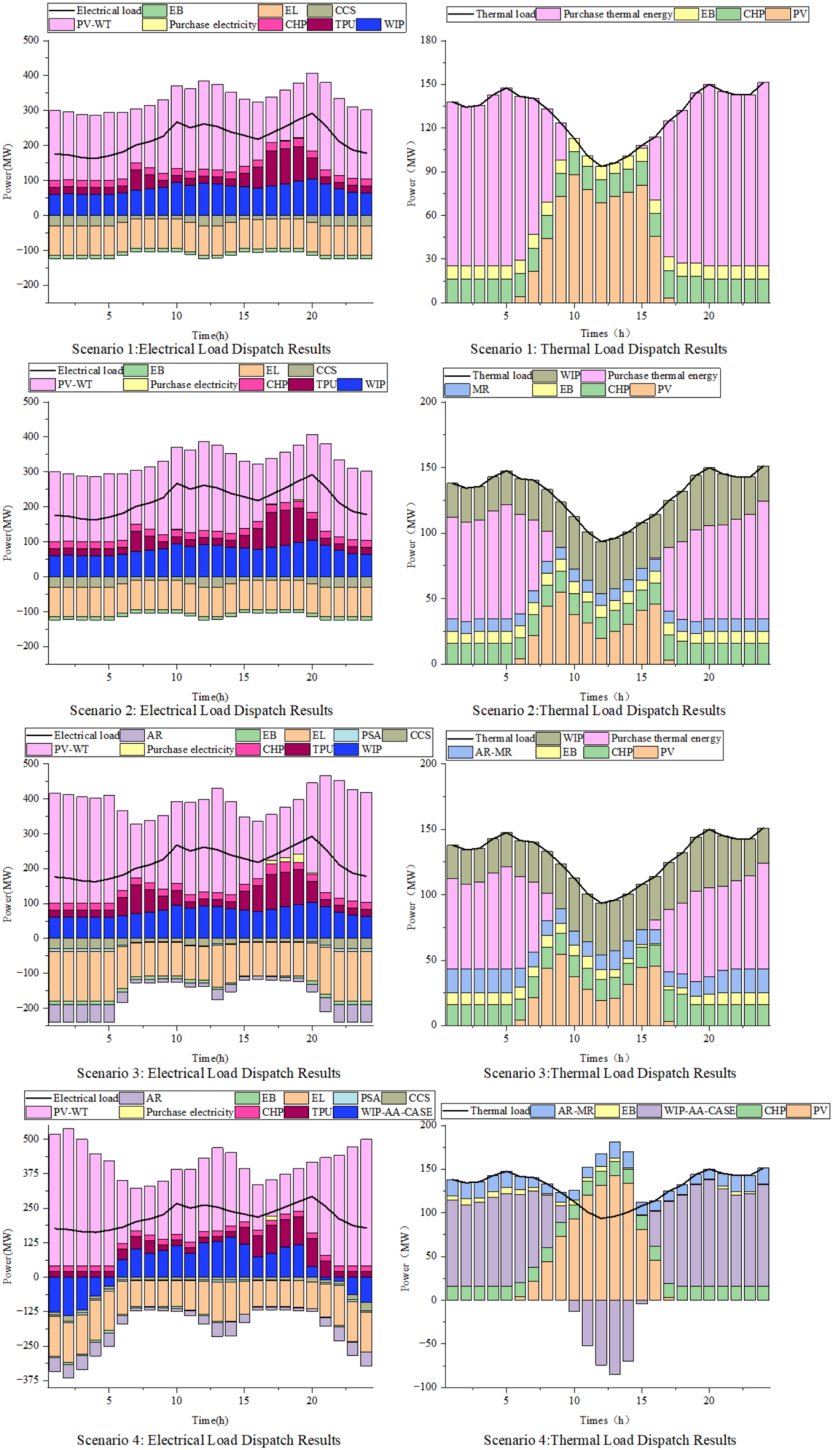
Scenario 1–4 Dispatch Results

Figure 7 shows the scheduling results for scenarios 1–4.

Scenario 2 incorporates WHB to recover waste heat from WIP and MR, resulting in a reduction of $21,445.86 in purchase heat cost, 360.28 t in carbon emissions, and $4,303.55 in carbon trading cost. This configuration achieves an overall cost reduction of 11.91%, thereby enhancing the economic performance of the system.

To address the wind and solar curtailment issues observed in Scenario 2, Scenario 3 introduces P2A technology to modify the thermal power unit operation, along with AR equipment to enhance renewable energy utilization and ammonia-based waste heat recovery for heat supply. Compared with Scenario 2, this approach reduces purchase heat cost by $2,809.43, carbon emissions by 13.83 t, and total cost by 11.45%, further improving both the economic viability and low-carbon characteristics of the system.

Scenario 4 introduces the AA-CAES to address the coordination challenges between WIP power generation and heat supply. This configuration achieves complete renewable energy accommodation, reducing purchase heat cost by $30,760.76, carbon emissions by 404.92 t, and total cost by 20.03% compared with Scenario 3. The integrated WIP-AA-CAES system flexibly balances power supply through compression energy storage and expansion-based power generation, effectively meeting both electricity and thermal load demands while significantly enhancing the economic and environmental performance of the IES.

### Impact of P2A on the System

To analyze the impact of P2A technology on the system, Scenario 2 and Scenario 3 are compared in terms of renewable energy curtailment, TPU operational status, and waste heat recovery by the WHB.

**Fig. 8:**
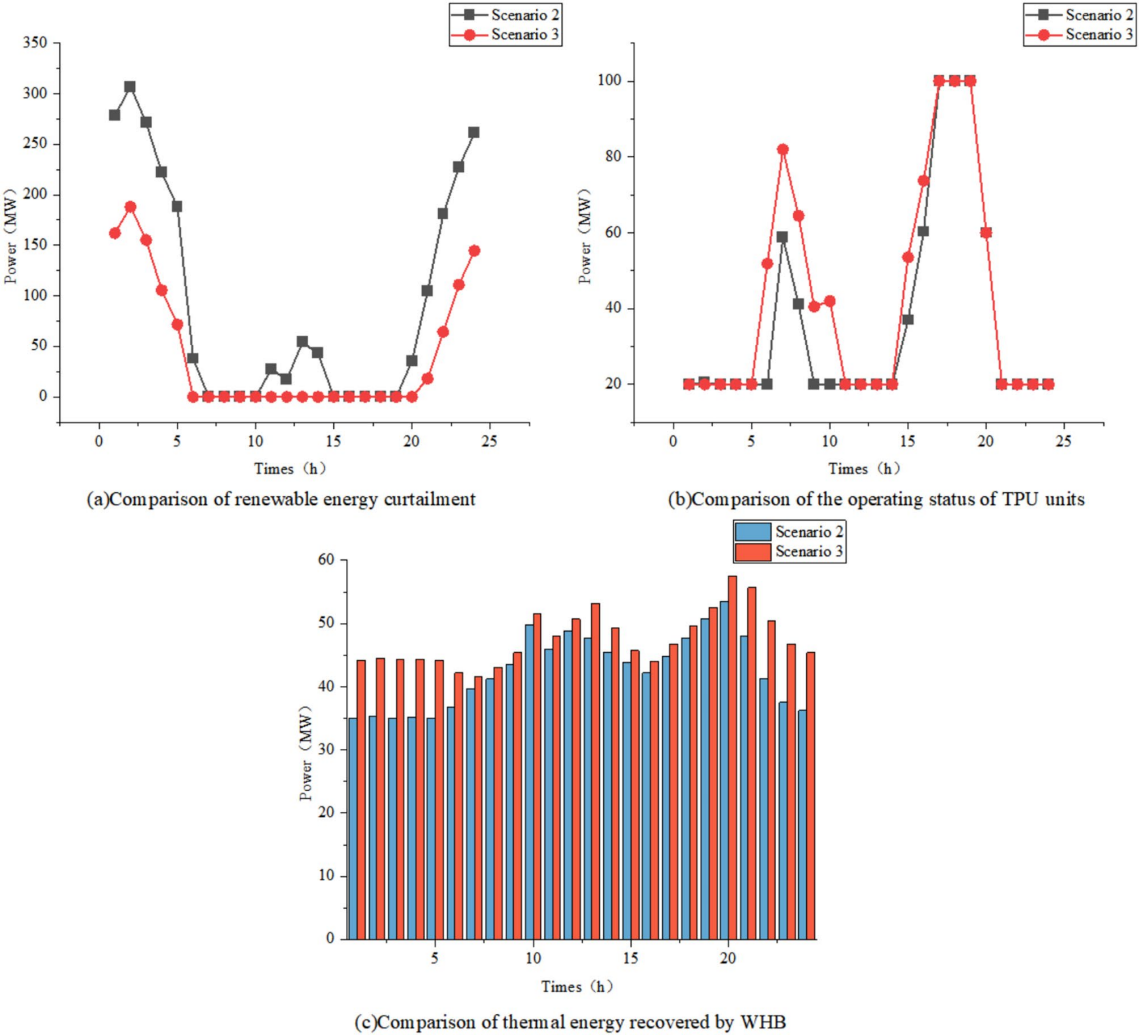
Comparison of data related to Scenario 2 and Scenario 3

As shown in Table 4, the introduction of P2A technology increases the renewable energy utilization rate from 68.02% to 85.49%, significantly enhancing renewable energy integration. Figures 8a and b reveal that during periods of 5:00–10:00 and 14:00–16:00, the thermal power unit output in Scenario 3 is higher than that in Scenario 2 due to insufficient renewable energy generation and the continuous operation requirement of the AR unit. However, as indicated in Table 4, despite the increased total thermal power output in Scenario 3, the partial substitution of coal combustion with ammonia results in lower coal consumption, thereby reducing both operational cost and carbon emissions. Furthermore, as illustrated in Fig. 8c, the WHB system recovers waste heat from the AR unit to satisfy a portion of the IES thermal energy demand, effectively alleviating heat supply pressure.

### Integration effect of AA-CASE

To analyze the impact of the AA-CASE device on the system, Scenario 3 and Scenario 4 are compared in terms of renewable energy curtailment, solar thermal utilization, and the operational status of the AR and WIP units.

**Fig. 9:**
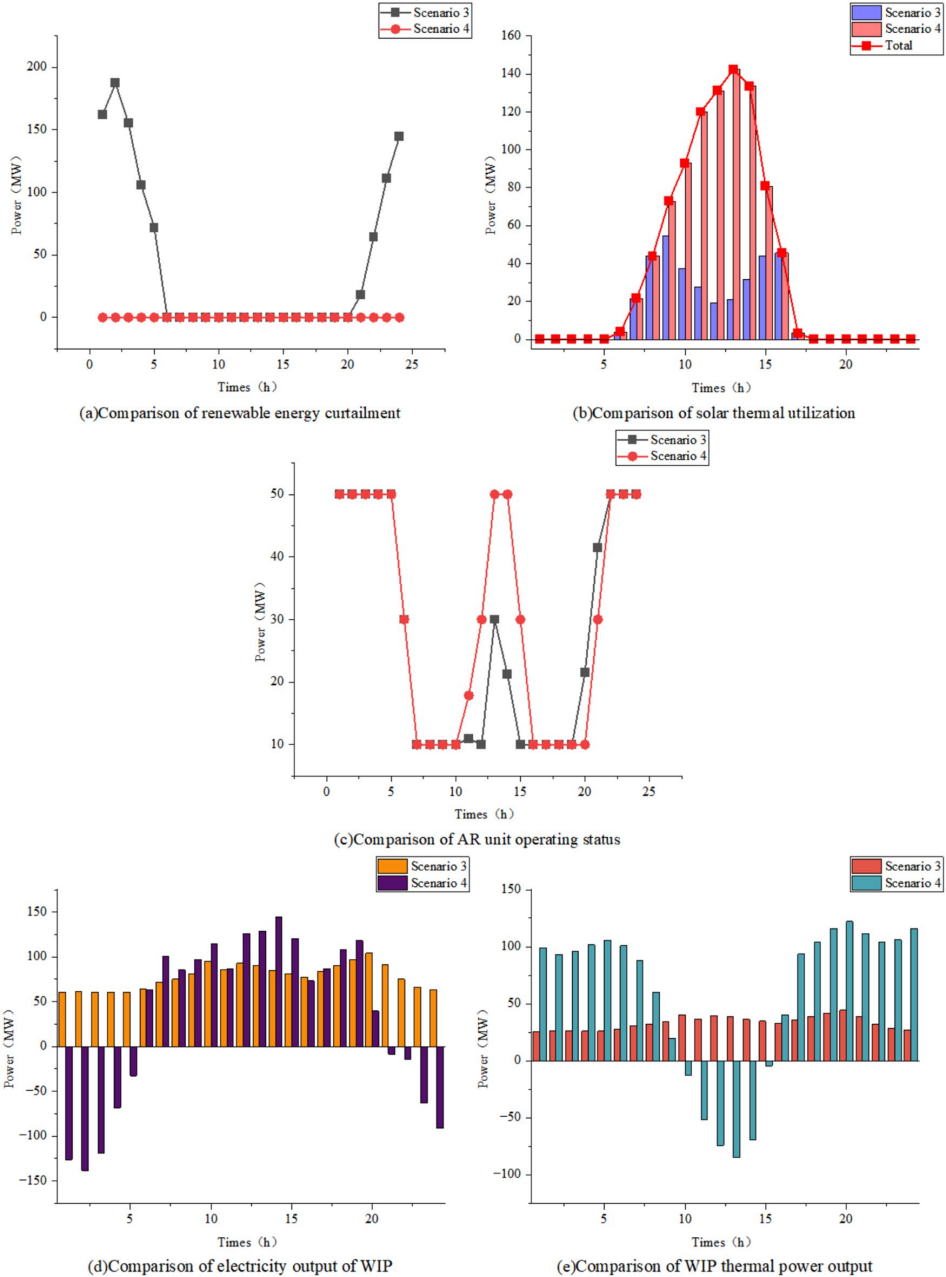
Comparison of data related to Scenario 3 and Scenario 4.

As illustrated in Figs. 9a and b, the introduction of the AA-CAES device in Scenario 4 enables complete utilization of renewable energy and solar-thermal energy, further enhancing overall energy efficiency. Combined with the analysis of Figs. 9d and e, during the 0:00–5:00 period characterized by low electrical load, high renewable energy generation, and high thermal demand, the AA-CAES stores excess renewable energy while simultaneously releasing thermal energy to meet the thermal load. During the 10:00–16:00 period, when thermal load is relatively low and solar-thermal generation is abundant, the AA-CAES absorbs heat for expansion-based power generation, thereby improving solar-thermal utilization and supplying the generated electricity to both the electrical load and AR equipment. As shown in Fig. 9c, Scenario 4 exhibits higher ammonia production during 10:00–16:00 because the AA-CAES absorbs heat for expansion and power generation to supply the AR equipment during this period. Conversely, ammonia production decreases during 19:00–21:00 when thermal load peaks, as the AA-CAES primarily operates in compression mode to store electricity and release heat, consequently reducing AR equipment output. In summary, the integration of AA-CAES enables temporal decoupling of electrical and thermal energy, significantly improving system economics and energy utilization efficiency.

### Impact of compressor inlet temperature on AA-CAES performance and IES

#### Impact on AA-CASE performance

**Fig. 10:**
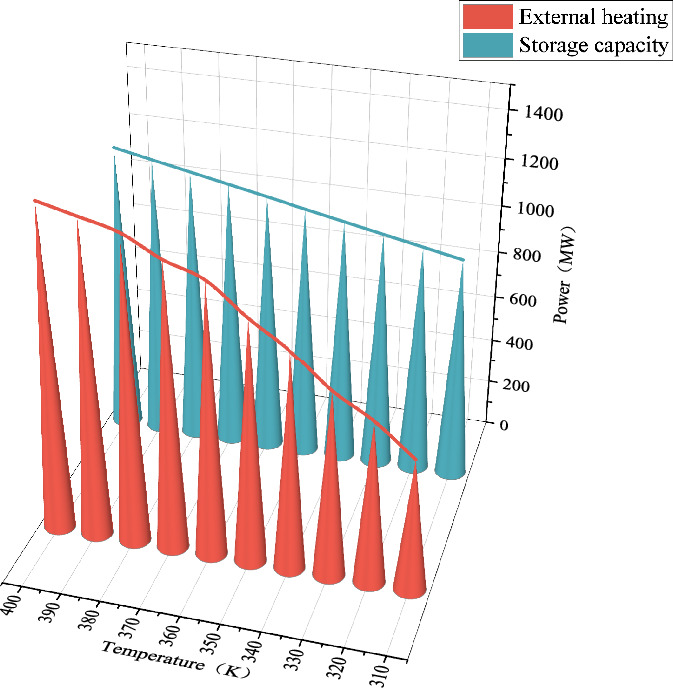
Impact of temperature on the performance of WIP-AA-CAES integrated model.

Scenario 4 is adopted as the baseline scenario, with the air temperature at the compressor inlet varied within the range of 310 K to 400 K to analyze the system performance variations. Figure 10 illustrates the impact of inlet air temperature on the performance of the WIP-AA-CAES integrated system.

In order to further analyze the trend of AA-CASE’s external heating and storage capacity with temperature, its related mathematical model is analyzed, and the related equations are as follows:

24 $$\left\{ \begin{gathered} P_{ch\arg e,e,t}^{{\text{AA - CASE}}} \left( - \right) = M_{ch\arg e,t} \left( \uparrow \right)\frac{\gamma }{\gamma - 1}R_{g} \sum\limits_{i = 1}^{{n_{1} }} {\frac{{T_{ch\arg e,in,i}^{{\text{AA - CASE}}} \left( \downarrow \right)}}{{\eta_{ch\arg e,i} }}\left( {\left( {\beta_{ch\arg e,i} } \right)^{{\frac{\gamma }{\gamma - 1}}} - 1} \right)} \hfill \\ P_{ch\arg e,h,t}^{{\text{AA - CASE}}} \left( \downarrow \right) = P_{ch\arg e,e,t}^{{\text{AA - CASE}}} \left( - \right)\frac{{\xi c_{air} \left( {\frac{{\left( {\beta_{ch\arg e,i} } \right)^{{\frac{\gamma }{\gamma - 1}}} - 1}}{{\eta_{ch\arg e,i} }} + 1 - \frac{{T_{cold} }}{{T_{ch\arg e,in,i}^{{\text{AA - CASE}}} \left( \downarrow \right)}}} \right)}}{{\frac{\gamma }{\gamma - 1}\frac{{R_{g} }}{{\eta_{ch\arg e,i} }}\left( {\left( {\beta_{ch\arg e,i} } \right)^{{\frac{\gamma }{\gamma - 1}}} - 1} \right)}} \hfill \\ Q_{t} \left( \uparrow \right) = \frac{{R_{g} \gamma T^{{{\mathrm{ST}}}} }}{{V^{{{\mathrm{ST}}}} }}\left( {M_{ch\arg e,t} \left( \uparrow \right)} \right) - \left( {\nu + \kappa \left| {M_{ch\arg e,t} \left( \uparrow \right)} \right|^{0.8} } \right)\left( {T^{{{\mathrm{ST}}}} - T_{wall}^{{{\mathrm{ST}}}} } \right) \hfill \\ \end{gathered} \right.$$

The electric power of the $P_{ch\arg e,e,t}^{{\text{AA - CASE}}}$ compressor remains unchanged, the $T_{ch\arg e,in,i}^{{\text{AA - CASE}}}$ compressor inlet air temperature decreases, the thermal power released by $M_{ch\arg e,t}$ compression decreases, the mass of air flowing into the compressor from the $T_{ch\arg e,in,i}^{{\text{AA - CASE}}}$ increases, and the increase in the mass of air increases the rate of change of the air pressure in the $Q_{t}$ storage chamber, and the maximum pressure in the storage chamber is a constant value, so the stored electric energy decreases. In summary, if the compressor inlet temperature is lowered, the external heat supply and storage capacity of AA-CASE will decrease, which is consistent with the simulation results.

#### Impact on IES

**Fig. 11:**
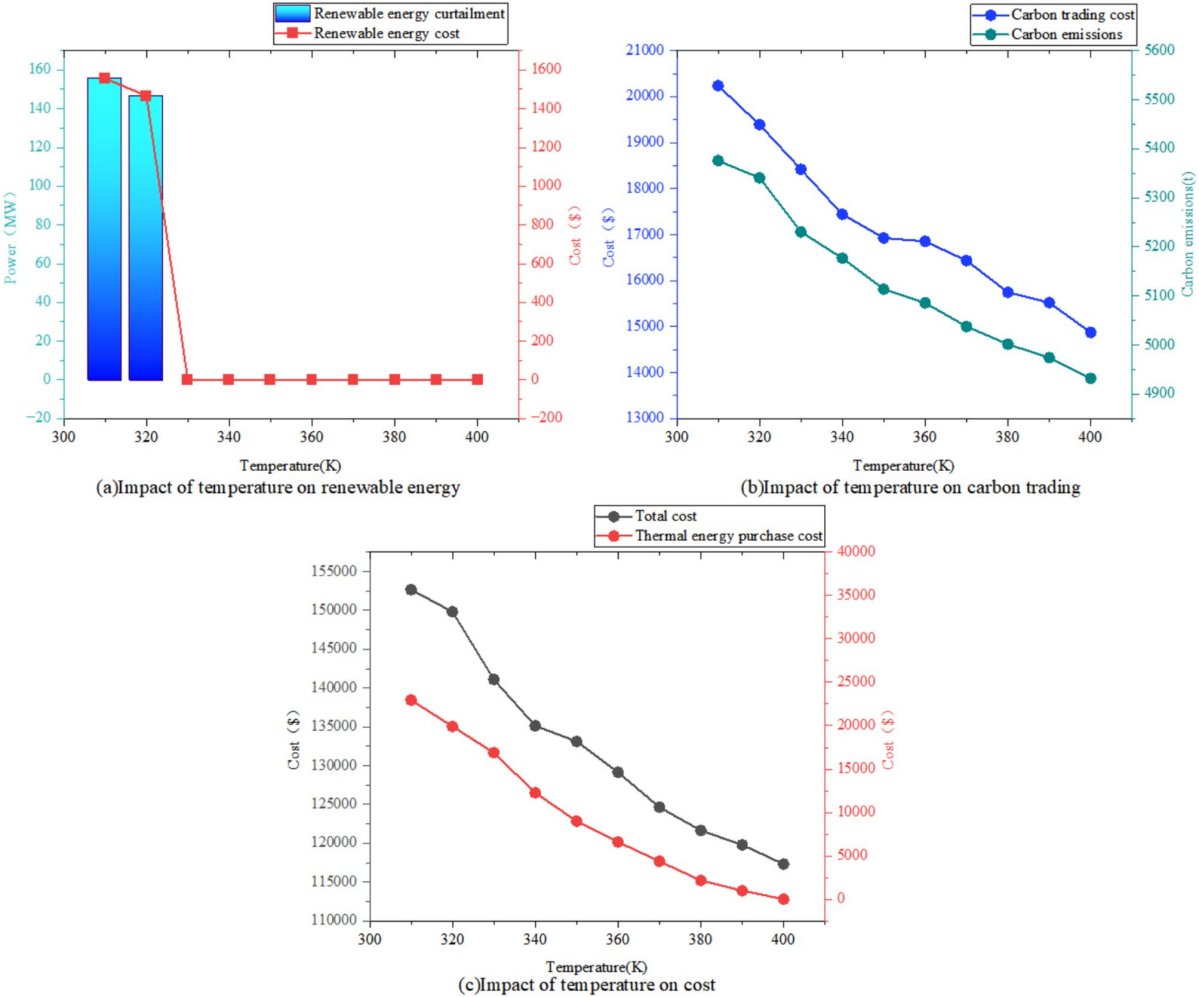
The Impact of Temperature on IES

Figure 11 illustrates the impact of compressor inlet temperature on renewable energy utilization, carbon trading cost, and total system cost of the IES. As the compressor inlet temperature increases, the integrated WIP-AA-CAES system enhances both external heat supply capacity and energy storage capacity, thereby improving renewable energy utilization. This improvement reduces purchased heat requirements and associated carbon emissions, consequently decreasing carbon trading cost and overall system cost.

## Conclusion.

Comparative analysis of the four scenarios investigated in this study demonstrates that the proposed synergistic model, which integrates WIP with P2G technology while incorporating waste heat recovery, P2A technology, and AA-CAES, effectively reduces carbon emissions, enhances renewable energy utilization, and improves system economics. The improved Particle Swarm Optimization algorithm efficiently solves the optimization model and yields superior scheduling strategies. The key findings are summarized as follows:

1. The WHB recovers waste heat from WIP and MR equipment, achieving a carbon emission reduction of 360.28 t and an 11.91% decrease in total cost. The P2A technology retrofits thermal generation units to reduce coal consumption, further reducing carbon emissions by 13.83 t and total cost by 11.45%, thereby realizing both economic and low-carbon operation of the IES.
2. Through the combination of AA-CASE and WIP, it realizes the complete consumption of renewable energy, and its compression storage and release of heat way to broaden the heat supply channel and expansion power generation and absorption of heat way to realize the use of electricity across the time, and the cost of purchasing heat has been reduced by 30,760.76$, and the carbon emission has been reduced by 404.92t, and the total cost has been reduced by 20.03%.
3. Heating the compressor inlet air effectively alleviates the heating demand of the IES and improves its economic performance. As the compressor inlet air temperature increases, the integrated WIP-AA-CAES model enhances external heat supply, thereby reducing purchase heat cost and carbon trading cost, ultimately lowering the total operational cost.
4. The PSO algorithm is enhanced through dynamic adjustment of inertia weights and learning factors, combined with a local exchange strategy. These improvements accelerate convergence and enable the algorithm to reach superior optimal solutions.

## Supplementary Information

Supplementary Information.

## Data Availability

Relevant data of this study are available from the corresponding author upon reasonable request.
