# Supplementary material for: Optimized scheduling of integrated energy systems considering waste-to-power plants and advanced adiabatic air compression energy storage machines
Source: Sci Rep. 2026 Feb 10;16:8041. doi: 10.1038/s41598-026-37485-z (PMC12957449; doi:10.1038/s41598-026-37485-z)
Supplement: Supplementary file 1 — Supplementary Information. [file 41598_2026_37485_MOESM1_ESM.docx]

Table A. 1Unit Parameters

| Unit | Parameter | | Unit |
| --- | --- | --- | --- |
| WIP | $M_{all}^{\text{WIP}}$ | 5000 | t |
|  | $\eta_{e}^{\text{WIP}},\eta_{h}^{\text{WIP}}$ | 0.5/0.4 | MWH/t |
|  | $\eta_{v}^{\text{WIP}}$ | 4000 | m^3^/t |
|  | $P_{e,\min}^{\text{WIP}},P_{e,\max}^{\text{WIP}}\text{，}P_{e,ramp}^{\text{WIP}}$ | 80/160/40 | MW |
| CCS | $P_{\min}^{\text{CCS}},P_{\max}^{\text{CCS}}\text{，}P_{e,ramp}^{\text{CCS}}$ | 10/30/10 | MW |
|  | $\eta_{\text{CO}_{2}}$ | 0.9 | t/MWH |
| PSA | $P_{\min}^{\text{PSA}},P_{\max}^{\text{PSA}}\text{，}P_{e,ramp}^{\text{PSA}}$ | 4/13/3 | MW |
|  | $\eta_{\text{N}_{2}}$ | 1 | t/MWH |
| EL | $P_{\min}^{\text{EL}},P_{\max}^{\text{EL}}$ | 0/150 | MW |
|  | $\eta^{\text{EL}}$ | 0.85 |  |
| MR | $P_{\min}^{\text{MR}},P_{\max}^{\text{MR}}\text{，}P_{H_{2},ramp}^{\text{MR}}$ | 20/50/10 | MW |
|  | $\eta^{\text{MR}}$ | 0.7 |  |
| AR | $P_{e,\min}^{\text{AR}},P_{e,\max}^{\text{AR}}\text{，}P_{e,ramp}^{\text{AR}}$ | 10/50/20 | MW |
|  | $\eta^{\text{AR}}$ | 1/7.4 | t/MWH |
| TPU | $P_{\min}^{\text{TPU}},P_{\text{max}}^{\text{TPU}}\text{，}P_{ramp}^{\text{TPU}}$ | 20/100/40 | MW |
|  | $\eta_{\text{CO}_{2}}^{\text{TPU}}$ | 2 | t |
|  | a | 0.0013 | t/MW^2^ |
|  | b | 0.24 | t/MW |
|  | c | 16 | t |
| CHP | $P_{e,\min}^{\text{CHP}},P_{e,\max}^{\text{CHP}}\text{，}P_{e,ramp}^{\text{CHP}}$ | 20/100/20 | MW |
|  | $\eta_{e}^{\text{CHP}},\eta_{h}^{\text{CHP}}$ | 0.4/0.8 |  |
| EB | $P_{h,t}^{\text{EB}},P_{e,t}^{\text{EB}}\text{，}P_{e,ramp}^{\text{EB}}$ | 1/10/1 | MW |
|  | $\eta^{\text{EB}}$ | 0.92 |  |
| WHB | $P_{h,\min}^{\text{WHB}}\text{，}P_{h,\mathrm{m}\text{ax}}^{\text{WHB}}$ | 10/80 | MW |
|  | $\eta_{h}^{\text{WHB,WIP}},\eta_{h}^{\text{WHB,AR}}$ | 0.4/0.85 |  |
| AA-CASE | $\begin{matrix} P_{\text{charge},e,\min}^{\text{AA-CASE}}\left(P_{\text{discharge},e,\min}^{\text{AA-CASE}}\right)\text{，} \\ P_{\text{charge,}e,\max}^{\text{AA-CASE}}\left(P_{\text{discharge},e,\max}^{\text{AA-CASE}}\right) \end{matrix}$ | 0/200 | MW |
|  | $n_{1},n_{2}$ | 2 |  |
|  | $\beta_{\text{charge},i},\beta_{\text{discharge},i}$ | 2.75/2.4 |  |
|  | $T_{\text{charge},in,i}^{\text{AA-CASE}},T_{\text{discharge},in,i}^{\text{AA-CASE}}$ | 400/532 | K |
|  | $\eta_{\text{charge},i},\eta_{\text{discharge},i}$ | 0.85 |  |
|  | $Q_{0}^{\text{ST}},Q_{\min}^{\text{ST}},Q_{\max}^{\text{ST}}$ | 4/4/7.5 | MPa |
|  | $V^{\text{ST}}$ | 20000 | m^3^ |
|  | $T^{\text{ST}},T_{wall}^{\text{ST}},T_{cold},T_{hot}$ | 440/448/422/579 | K |
|  | $\nu,\kappa,\xi$ | 0.02356/0.0149/0.85 |  |
|  | $c_{air}$ | 1005 | J/（kg·K） |
|  | $H_{\min}^{\text{ST}},H_{\max}^{\text{ST}},H_{\text{change},\max}^{\text{ST}}$ | 20/320/100 | MWH |

Table A. 2parameters

| Unit | Parameter | Unit |
| --- | --- | --- |
|  | 10/25/30/35 | $/MWH |
|  | 30/80/2 | $/t |
|  | 0.5 | $/m^3^ |
| $E^{\text{WIP}},E^{\text{TPU}}$ | 1.53/0.4 | t/MWH |
| $\beta^{\text{CHP}},E^{\text{CHP}}$ | 0.4/0.45 | t/MWH |
| $\beta_{e}^{\text{BUY}},E_{e}^{\text{BUY}}$ | 0.35/0.4 | t/MWH |
| $\beta_{h}^{\text{BUY}},E_{h}^{\text{BUY}}$ | 0.3/0.5 | t/MWH |
|  | 150000/50000/30000/150000/200000/100000 | $/MW |
|  | 0.003 | $/m^3^ |
|  | 4140 | $/MWH |
| $\alpha_{\text{NH}_{3}}$ | 1.6 | MW/t |
| Calorific value of H2 | 12.855 | MJ/m^3^ |
| Calorific value of CH4 | 36 | MJ/m^3^ |
| Calorific value of coal | 23022 | kJ/kg |
| Calorific value of NH3 | 18720 | kJ/kg |
| $\rho_{\text{CO}_{2}},\rho_{\text{N}_{2}},\rho_{\text{NH}_{3}}$ | 1.977/1.36/0.71 | kg/m^3^ |
